# Identification of PsbS binding proteins in *Arabidopsis thaliana* leaf chloroplasts under high light using TurboID-based proximity labeling

**DOI:** 10.3389/fpls.2025.1705804

**Published:** 2026-01-20

**Authors:** Yuwei Jiao, Yanhui Dou, Lihua Wang, Xin-Guang Zhu, Huiqiong Zheng

**Affiliations:** 1 Center for Excellence in Molecular Plant Sciences, Chinese Academy of Sciences, Shanghai, China; 2 University of Chinese Academy of Sciences, Beijing, China

**Keywords:** *Arabidopsis thaliana*, NPQ, photosynthesis, proximity labeling proteomics, PsbS

## Abstract

Photosystem II Subunit S (PsbS) is a critical regulator of non-photochemical quenching (NPQ), which is a protective mechanism triggered to dissipate excess light energy as heat and prevent photodamage. However, the molecular basis of how PsbS interact with partner proteins to regulate NPQ remains unclear. In this study, we employed proximity labeling to identify PsbS interaction proteins *in situ* in living cells of Arabidopsis leaves via biotinylation during NPQ. Arabidopsis plants stably expressing PsbS constructs fused to proximity labeling enzyme TurboID were generated and the biotinylated proteomes were analyzed by liquid chromatography-mass spectrometry. The interactomes of PsbS under dark and under light were generated, which not only confirmed several known PsbS-interacting proteins, such as Lhcb1.3, Lhcb3, and Lhcb4.2, but also identified many novel binding proteins. Interestingly, most of these protein interactions of PsbS were unaffected by light, which suggest that PsbS might influence the NPQ through conformational changes without a large physical migration within thylakoid membrane. Analyses of the interactomes also show a few proteins enhanced (such as TLP18.3) or some proteins inhibited (such as ZEP) under the high light, suggesting that the NPQ and repair process after photoinhibition might be coordinated.

## 1 Introduction

Light plays a dual role in photosynthesis, serving as both the energy source and a potential stress-inducing factor. Under appropriate light intensity, the photochemical efficiency can be effectively promoted, when plants are exposed to excessive high light (HL) or fluctuating light, photodamage can be triggered due to the generation of reactive oxygen species (Apel and Hirt, 2004). To cope with this challenge, plants evolved a non-photochemical quenching (NPQ) mechanism. This mechanism can dissipate the excess excitation energy as heat before reaching photosystem center. There are different components of NPQ, i.e. qE, qH, qI, qM, qT, and qZ, each accomplishing energy dissipation at different time scales, qE is the most prominent form in higher plants and can be activated to induce deexcitation of chlorophylls within a few seconds to minutes (Lu et al., 2022). The induction of qE depends on three key components that act in concert: (1) acidification of the thylakoid lumen (ΔpH); (2) accumulation of zeaxanthin through the xanthophyll cycle; and (3) activation of the PsbS protein to transmit feedback signals for photoprotection to the light-harvesting complexes (LHC) - photosystem II (PSII) network, establishing quencher states for dissipation of excess light energy (Li et al., 2000, 2002; Chen et al., 2025).

PsbS is a 22-kDa transmembrane protein with four transmembrane helices and two amphipathic helical stretches at the luminal side of the thylakoid membrane, which mediates qE through pH-dependent structural rearrangements in PSII LHCs (Valencia and Pandit, 2024). Two lumen-exposed glutamate residues as proton sensors are essential for the function of PsbS in establishing NPQ (Fan et al., 2015; Liguori et al., 2019). It has been suggested that monomerization of PsbS occurs at low pH in thylakoid membranes, where PsbS may interact with various partners. The PSII core is surrounded by the LHC minor antennas (CP29, CP26 and CP24), which can bind the peripheral antennas LHCII (forming PSII-LHCII). A model suggests that PsbS is associated with the PSII core proteins in the dark, while increased interaction of PsbS with LHCII occurs in the HL conditions, accompanied by the detachment and/or aggregation of LHCII proteins (Valencia and Pandit, 2024). This hypothesis is supported by several previous experiments using chemical crosslinking and immunolabeling (Correa-Galvis et al., 2016), and magnetic-bead-linked antibody pull-down (Sacharz et al., 2017; Teardo et al., 2007). However, NPQ-specific interaction partners of PsbS in the thylakoid membrane are still unknown due to the rapid dynamic process of qE involving transient interactions of PsbS with partners (Caffarri et al., 2009; Gerotto et al., 2015), which could not be captured by traditional protein chemical crosslinking and affinity purification analysis. A new method, which can assess the weak or transient interactome network of PsbS during NPQ, is needed.

Recent development in proximity labeling (PL) technologies circumvent these limitations through *in vivo* biotinylation of adjacent proteins. TurboID, an engineered biotin ligase enabling rapid labeling (<10 min) with minimal cellular toxicity (Branon et al., 2018), has shown particular efficacy in plant membrane proteomics (Arora et al., 2020). By employing PsbS-TurboID fusion lines in Arabidopsis, we systematically mapped interactome dynamics of PsbS under the dark and the HL conditions. Our analysis not only confirms established partners (such as, Lhcb1 and Lhcb4) but reveals previously unidentified interactors connecting PsbS to xanthophyll cycle (ZEP) and thylakoid membrane remodeling (TLP18.3). Analyses of these interactomes provide new insights into the location of PsbS and the mechanism through which PsbS influence NPQ.

## 2 Materials and methods

### 2.1 Plant materials and growth conditions

*Arabidopsis thaliana* materials used in this study included: Wild-type (WT, Col-0 ecotype), PsbS-defective mutant *npq4* (Salk_095156), zeaxanthin epoxidase (ZEP)-defective mutant *npq2* (Salk_027326C), and thylakoid lumen protein 18.3 kDa (TLP18.3)-defective mutant *tlp18.3* (Salk_050942). Seeds were surface-sterilized with 75% (v/v) ethanol for 15 min, washed multiple times with sterile water, and then incubated at 4°C in the dark for 48–72 h. Germination was initiated on 1/2Murashige and Skoog basal medium supplemented with 1% (w/v) sucrose under controlled conditions: 16-h photoperiod (100 μmol m^-2^. s^-1^ photosynthetic photon flux density), 22°C, and 65% relative humidity. After 4~14 days of growth, seedlings were transplanted to soil and maintained in a greenhouse with identical growth conditions.

### 2.2 Construction of transgenic plants

By amplifying the TurboID coding sequence from *3xHA-TurboID-NLS_pCDNA3* (Branon et al., 2018) with primers TbID-F1/R1 (retaining the 3xHA tag) and cloning it into *pHB_35Spro::MCS-eGFP*, *pHB_35Spro::TurboID-eGFP* construct was generated. For the PsbS-TurboID-eGFP construct, Arabidopsis PsbS gene *NPQ4* (AT1G44575) and *TurboID* gene were amplified with specific primers and inserted into the same vector via homologous recombination. A control construct (*pHB_35Spro::PTP-TurboID-eGFP*) used the PsbS chloroplast transit peptide (PTP). Here we used the N-terminal PTP of PsbS to help the TurboID fusion control protein (PTP-TurboID-eGFP) to be transferred to chloroplasts and be inserted into the thylakoid membrane, where the PTP was cleaved off, as the PTP in PsbS-TurboID-eGFP. By this process, the subcellular localization pattern of control protein (PTP-TurboID-eGFP) was similar to the fusion protein PsbS-TurboID-eGFP. In addition, the control PTP-TurboID-eGFP should show an expression level similar to that of the PsbS-TurboID-eGFP protein (**Supplementary Figure S2**). All primers used in this study are listed in **Supplementary Table S5**. All constructs were validated by DNA sequencing.

Three PL vectors were introduced into *Agrobacterium tumefaciens* GV3101 for Col-0 transformation via floral dip (Clough and Bent, 1998). T1 transformants were selected on1/2MS medium with 50 mg/L hygromycin and 200 mg/L timentin, stratified at 4°C for 48 h, then grown under 16-h light/8-h dark at 22°C. Hygromycin-resistant seedlings were transplanted to soil. T2 seeds were collected and screened on selection medium to identify lines with a 3:1 segregation ratio (resistant: sensitive), indicating single-locus insertion. T3 seeds from T2 plants showing 100% resistance were considered homozygous and used for subsequent experiments.

### 2.3 qRT-PCR analyses

Total RNA was isolated from Arabidopsis seedlings using KK Fast Plant Total RNA Kit (ZOMANBIO, #ZP405K). RNA quality/concentration was verified by NanoDrop. First-strand cDNA was synthesized from 1 μg of total RNA with PrimeScript RT Kit (Takara). Gene-specific primers were designed by Primer-BLAST and synthesized commercially. qPCR was performed on a Lightcycler 96 (Roche Applied Science) using TB Green Premix Ex Taq II (Takara). Each reaction contained cDNA, primers, and premix. Cycling conditions were 95°C for 30 s, followed by 40 cycles of 95°C for 5 s and 60°C for 30 s. Relative gene expression was calculated using the 2-ΔΔCt method with the constitutive housekeeping gene *ADENINE PHOSPHORIBOSYL TRANSFERASE 1* (*APT1*, At1g27450) as the reference gene. Experiments were conducted at least three times with similar results. The primers used in this study are listed in **Supplementary Table S5**.

### 2.4 Biotin treatment and protein extraction for TurboID

For TurboID biotin treatment, approximately 20 mg 5-day old seedlings were immersed in 50 µM biotin in 12-well plates, incubated under high light (600 μmol·m^−^²·s^−^¹) or kept under the dark at 22°C for 3h. Optimization of biotin concentrations (0, 1, 10, 50, 100 and 250 μM, respectively) and durations (0, 15, 30, 60, 120 and 180 min, respectively) were tested. After the reactions were quenched in ice-water, samples were then dried, flash-frozen in liquid nitrogen, or stored at -80°C.

For protein extraction, frozen tissues were ground in liquid nitrogen, homogenized with 100 μL extraction buffer (50 mM Tris-HCl pH7.5, 150 mM NaCl, 5 mM MgCl_2_, 10% glycerol, 0.1% NP-40, 0.5 mM DTT, 1 mM PMSF, 1× protease inhibitors), and incubated on ice for 30 min with vortexing. After centrifugation at 13,000×g for 20 min at 4°C, supernatants were mixed with 5× SDS loading buffer and denatured at 98°C for 10 min. Immunoblotting was performed as described (Branon et al., 2018) using anti-GFP and streptavidin-HRP to detect biotinylated proteins, with Coomassie staining for loading control.

### 2.5 Preparation of TurboID samples

For TurboID sample preparation, 5-day old Arabidopsis seedlings (0.8 g per replicate, three biological replicates) were immersed in 50 µM biotin under high light (HL, 600 μmol·m^−^²·s^−^¹) or under the dark (D) at 22°C for 3 h, washed three times with ice-cold water (1 min each), dried, flash-frozen in liquid nitrogen, and stored at -80°C.

Frozen tissues were ground in liquid nitrogen, homogenized with 4 mL extraction buffer, and centrifuged at 17,000×g for 15 min at 4°C. After loading the sample onto the Zeba™ desalting column, desalting was performed by centrifugation. Streptavidin magnetic beads (120 µL) were washed 3× with protein extraction buffer, incubated with desalted proteins at 4°C for 12–16 h with rotation. After incubation, beads were washed sequentially with extraction buffer, 50 mM KCl, 0.1 M Na_2_CO_3_, and 8 M urea, then proteins were eluted by heating in laemmli buffer.

### 2.6 On-bead trypsin digestion of biotinylated proteins

DTT (with a final concentration of 10 mM) was added to each sample and mixed at 600 rpm for 1.5 h at 37°C. Samples were then alkylated with 20 mM iodoacetamide (IAA) in the dark for 30 min, transferred to 10-kDa MWCO filters, and washed sequentially with UA buffer (8 M urea in 100 mM Tris-HCl, pH 8.0) and 25 mM NH_4_HCO_3_. Trypsin digestion (1:50 enzyme-to-protein ratio) was performed at 37°C for 15–18 h. Peptides were collected by centrifugation, desalted using C18 SPE cartridges (Empore™), vacuum-concentrated, and reconstituted in 40 μL 0.1% formic acid. Peptide concentration was determined by UV absorbance at 280 nm.

### 2.7 liquid chromatography-tandem mass spectrometry analysis

LC-MS/MS analysis was performed using a Q Exactive mass spectrometer (Thermo Scientific) coupled to an Easy-nLC 1200 system. Peptides were loaded onto a C18 trap column (Acclaim PepMap100, 100 μm × 2 cm) and separated on a C18 analytical column (75 μm × 10 cm, 3 μm resin) with a 60-min gradient of buffer B (84% acetonitrile/0.1% formic acid) at 300 nL/min. Mobile phases were buffer A (0.1% formic acid) and buffer B.

Mass spectrometry used positive ion mode with data-dependent acquisition of top 20 precursor ions (m/z 300-1800) for HCD fragmentation. Full MS scans were at 60,000 resolution (m/z 200), AGC target 1×10^6^, max injection time 50 ms with an automatic gain control (AGC) target of 1×10^6^ ions. HCD spectra was set to 15,000 at m/z 200 and isolation width was 1.5 m/z, 30 eV collision energy. Dynamic exclusion was set to 30 s with an underfill ratio of 0.1%, and peptide recognition mode was enabled. The raw data were uploaded successfully to the ProteomeXchange (proteomecentral.proteomexchange.org/) with accession number PXD069971.

### 2.8 MS data analysis

Raw MS data were processed using MaxQuant (v1.6.14) for protein identification and label-free quantitation (LFQ). Database searches were performed against the UniProtKB *Arabidopsis thaliana* database with trypsin digestion allowing up to two missed cleavages. The fixed modification was set as biotinylation of lysine. The mass tolerance for the main search was 6 ppm, 20 ppm for the first search, and 20 ppm for the MS/MS spectra. The target-decoy approach with reversed sequences controlled false discovery rates at ≤1% for both proteins and peptides.

**Figure 1:**
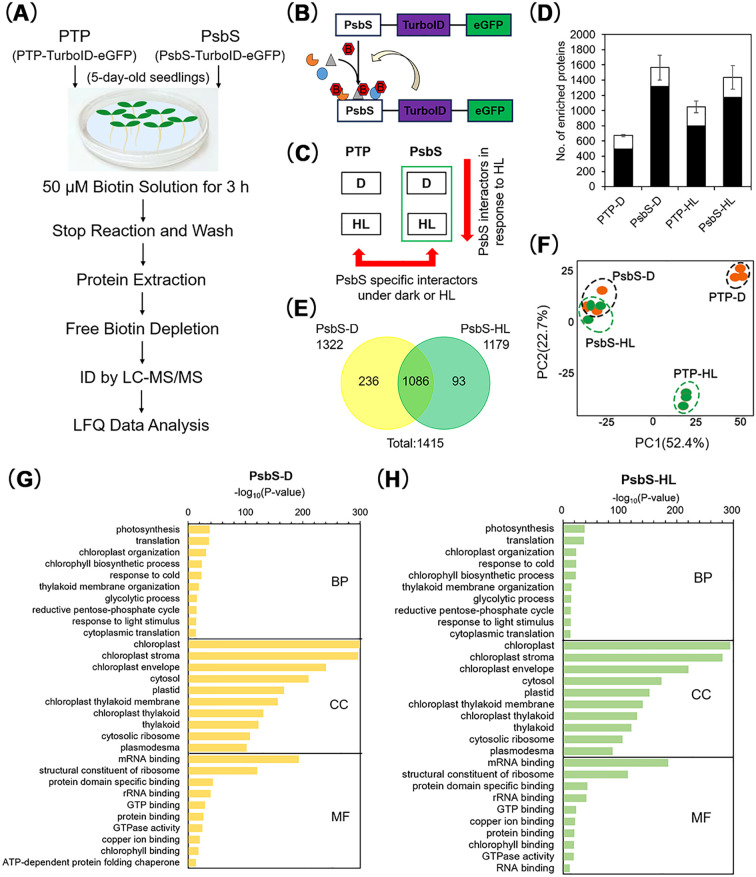
Proximity-based interactome of PsbS under dark and high light conditions. **(A-C)** Experimental procedures, principles, and data analysis processes of proximity-based (PL) proteomics. **(D)** Label-free quantification (LFQ) analysis via mass spectrometry identified the total number of biotinylated proteins in PsbS-TurboID-eGFP (#18) and PTP-TurboID-eGFP (#19) plants under dark and high light conditions. Three biological replicates were performed for each sample group; the total protein count represents the average of replicates with consistent identification. **(E)** Venn diagrams separately depicting the biotinylated proteomes identified in PsbS-TurboID-eGFP (#18) transgenic plants under dark (PsbS-D) and high light (PsbS-HL) conditions. The overlapping and unique proteins were summarized from three biological replicates; proteins were considered consistently identified only if detected in all replicates. **(F)** Principal component analysis (PCA) of the PsbS proximity-labeled proteome. PCA revealed significant differences between biotinylated proteins identified in PsbS-TurboID-eGFP (#18) transgenic plants under dark (PsbS-D) and high light (PsbS-HL) conditions compared to the non-specific binding controls (PTP-D and PTP-HL) expressing PTP-TurboID-eGFP (#19) under the same conditions. **(G, H)** Gene Ontology (GO) term enrichment analysis of biotinylated proteins identified in PsbS-TurboID-eGFP (#18) transgenic plants under dark (PsbS-D) and high light (PsbS-HL) conditions.

LFQ values were log_2_ transformed and only proteins with at least unique peptides and two spectral counts were considered identified in an individual sample. If a protein was not identified in a sample, the LFQ intensity was set to zero. To determine the cutoff for proteins enriched in PsbS-TurboID-eGFP samples, identified proteins were cross-referenced with true positive or false positive lists. The true positive lists were assembled using localization data from studies of the thylakoid in Arabidopsis. A total of four analyses were performed (**Figure 1C**), one for each enrichment metric (log_2_ (HL/D) using LFQ intensity and log_2_ (HL/D) using normalized spectral counts) in each sample. For each PsbS-TurboID-eGFP PL protein in every analysis, the log_2_(PsbS LFQ intensity/PTP LFQ intensity) <1 were classified as “non-specific” binding proteins (the cut-off), while those showing significant abundance in (log_2_ (PsbS LFQ intensity/PTP LFQ intensity)≥1, the false discovery rate (FDR) <0.05) under the dark or the HL conditions were designated “specific binding proteins”. The proteins above the cut-off of the in all 4 analyses are reported in **Supplementary Table S3**. Ontology (GO) enrichment analyses were conducted using the DAVID website (https://davidbioinformatics.nih.gov). Protein-Protein interaction (PPI) network was constructed by the STRING website (https://cn.string-db.org, minimum edge score≥0.7).

### 2.9 Split-luciferase complementation assay

The Split-LUC assay in *Nicotiana benthamiana* was performed according to the protocol established by Zhou et al. (2018) with minor modifications. For luciferase complementation, the PsbS transit peptide, firefly luciferase C-terminal, and PsbS CDS were recombined to form *35Spro::PTP-cLuc-PsbS*, with bait proteins (ZEP, TLP18.3) linked to luciferase N-terminal. For luciferase activity quantification, leaf samples were exposed to HL (600 μmol m^−^² s^−^¹) for 3 hours at 2 days post-inoculation (dpi) to induce cellular responses, followed by collection and uniform spraying with a 1 mM d-luciferin solution. Incubation was then performed under dark conditions for 10 minutes to allow substrate penetration. Luminescence signals were subsequently captured using a charge-coupled device imaging system (ChemiDoc, Bio-Rad), and three biological replicates (independent plants) were analyzed for each experimental condition.

### 2.10 Bimolecular fluorescence complementation

BiFC was performed as previously described (Wang et al., 2020). Briefly, constructs encoding nYFP-PsbS and cYFP-bait proteins (ZEP, TLP18.3) were co-transformed, and reconstituted YFP signals were detected. YFP signals (514 nm excitation, 500–535 nm emission) and chloroplast autofluorescence (488 nm excitation, 650–750 nm emission) were imaged under consistent photomultiplier gain and laser power. BiFC controls included empty vector combinations to assess background fluorescence. All images were acquired under consistent photomultiplier gain and laser power conditions.

### 2.11 NPQ measurements

NPQ phenotypes of 4~6-week-old Arabidopsis seedlings grown under control conditions (100 μmol photons m^−^² s^−^¹, 16 h light/8 h dark cycle) were assessed using a Dual-PAM-100 chlorophyll fluorescence monitoring system (Walz, Effeltrich, Germany) and chlorophyll fluorescence imaging was performed using a CF Imager (Technologica Ltd., UK) at room temperature. Prior to measurements, plants were dark-adapted for 2 hours to allow full relaxation of PSII and reset fluorescence parameters (e.g., Fo and Fm) to their dark-adapted states.

For induction kinetic experiments, actinic light intensity was set to 685 μmol photons m^−^² s^−^¹. PSII parameters were calculated as follows: Maximum quantum yield of PSII Y(II) = (Fm’ - Fo’)/Fm’; NPQ = (Fm - Fm’)/Fm’, where Fo represents the minimum fluorescence of dark-adapted leaves, Fm denotes the maximum fluorescence after a saturation pulse under dark adaptation, and Fm’ indicates the maximum fluorescence measured under actinic light illumination. All experimental data are presented as mean ± standard deviation from at least three independent biological replicates.

## 3 Results

### 3.1 Construction of PsbS-TurboID-eGFP transgenic plants for identifying PsbS binding proteins during non-photochemical quenching

**Figure 2:**
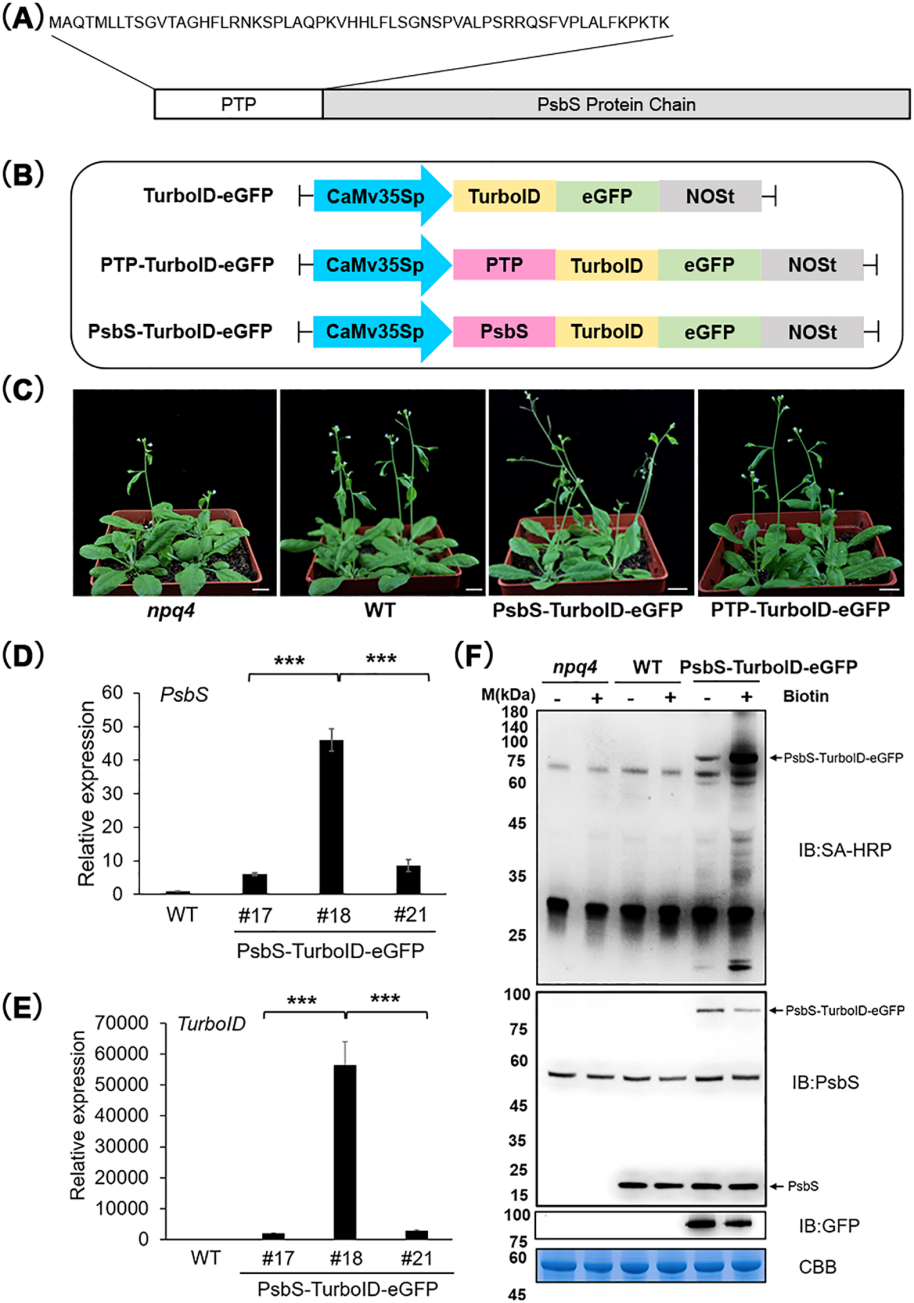
Construction and identification of PsbS-TurboID-eGFP and control transgenic plants. **(A)** Schematic diagram of the PsbS protein structure, showing a predicted 59-amino-acid protein transit peptide (PTP) at the N-terminus. **(B)** Schematic diagram of vector construction. *CaMV35Spro::TurboID-eGFP* (referred to as *TurboID-eGFP*) fusion vector was constructed by adding an eGFP tag to the C-terminus of TurboID. Then, the PTP of PsbS and the full-length gene of PsbS were amplified from Arabidopsis cDNA and fused to the N-terminus of TurboID-eGFP, thus constructing the *35Spro::PTP-TurboID-eGFP* and *35Spro::PsbS-TurboID-eGFP* fusion vectors (referred to as *PTP-TurboID-eGFP* and *PsbS-TurboID-eGFP*, respectively). **(C)** Phenotypes of 4-week-old *npq4*(PsbS-defective mutant), wild-type (WT, Col-0 ecotype), and transgenic plants PsbS-TurboID-eGFP(#18) and PTP-TurboID-eGFP(#19). Scale bar =1 cm. The gene *APT1* (AT1G27450) was used as an internal control. **(D, E)** Quantitative real-time reverse transcription polymerase chain reaction (qRT-PCR) was employed to detect the expression of PsbS **(D)** and TurboID **(E)** in transgenic PsbS-TurboID-eGFP plants. Data are presented as mean ± SD (n = 3). Asterisks indicate significant differences compared to WT (Student’s t-test, ***p < 0.001). **(F)** Western blot analysis was used to detect the expression of PsbS and TurboID in transgenic Arabidopsis. The seedlings of *npq4* mutant, WT, and PsbS-TurboID-eGFP Arabidopsis were treated with biotin (+, 50 μM, 1 h) or left untreated (-). Immunoblotting (IB) was performed using Streptavidin-Horseradish Peroxidase (SA-HRP), anti-PsbS, and anti-GFP antibodies to analyze the activity and expression level of PsbS-TurboID-eGFP. CBB: Coomassie Brilliant Blue. Molecular weight markers in kDa are shown on the left side.

PsbS is a nuclear-encoded chloroplast membrane protein and is first expressed in the cytoplasm. A PsbS protein transit peptide (PTP), which was predicted in the nitroxyl-terminal (N-terminal) with 59 amino acids (**Figure 2A**), is required for PsbS imports into the chloroplast. The PTP sequence was reported to be cleaved after PsbS imports the chloroplast (Niyogi et al., 2005). For the purpose of identifying the protein interactome of PsbS *in vivo* under the HL conditions, we employed TurboID-based PL, a technology capable of efficiently labeling proteins by TurboID within a 10-nanometer distance (Branon et al., 2018). Firstly, we constructed a *CaMV35Spro:: TurboID-eGFP* fusion vector (hereinafter referred to as TurboID-eGFP) by appending an eGFP tag to the C-terminus of TurboID (**Figure 2B**). Then, the cDNA of exclusive PTP and the coding region of PsbS with PTP of Arabidopsis were cloned and fused to the N-terminus of TurboID-eGFP, creating *35Spro::PTP-TurboID-eGFP* and *35Spro::PsbS-TurboID-eGFP* fusion sequences, respectively (hereinafter referred to as PTP-TurboID-eGFP and PsbS-TurboID-eGFP, respectively) (**Figure 2B**). Three types of these plasmids were transformed into Arabidopsis (wild-type, Col-0), and the independent transgenic lines that expressed TurboID-eGFP, PTP-TurboID-eGFP and PsbS-TurboID-eGFP were generated (**Figure 2C**). No differences in vegetative growth and development were observed among any of the transgenic lines in comparison with wild-type (WT) and *npq4* (**Supplementary Figure S1**) under the normal growth condition (**Figure 2C**). PsbS-TurboID-eGFP independent transgenic lines (#17, #18 and #21) were selected for further analysis. RT-PCR and immunoblotting confirmed expression of PsbS and TurboID in selected transgenic lines at transcriptional and translational levels (**Figures 2D-F**; **Supplementary Figure S2**).

### 3.2 Optimization of biotin proximity labeling conditions

**Figure 3:**
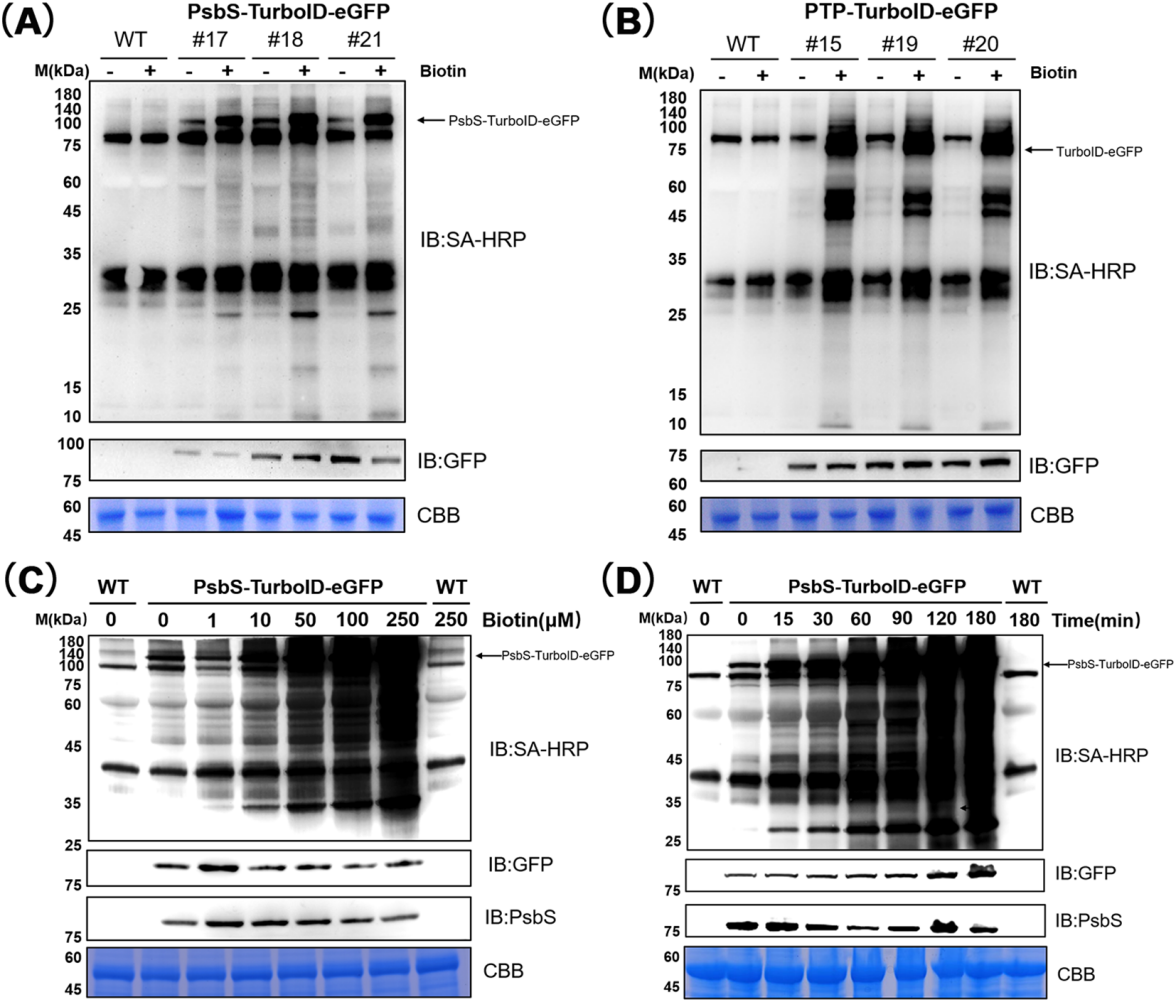
Optimization of proximity labeling experimental conditions. **(A)** Selected seedlings expressing PsbS-TurboID-eGFP (#17, #18, #21) and wild-type (WT, Col-0 ecotype) were treated with biotin (+, 50 μM, 1 h) or without biotin (–), followed by immunoblotting detection. **(B)** Selected seedlings expressing PTP-TurboID-eGFP(#15, #19, #20) and WT were treated with biotin (+, 50 μM, 1 h) or without biotin (–), followed by immunoblotting (IB) detection. **(C)** WT and PsbS-TurboID-eGFP (#18) were treated with biotin solutions of different concentrations for 1 hour. Immunoblotting was performed using Streptavidin-HRP (SA-HRP) and anti-GFP antibodies to detect the activity and expression level of PsbS-TurboID-eGFP. **(D)** WT and PsbS-TurboID-eGFP (#18) were treated with a 50 μM biotin solution for different durations. Immunoblotting was performed using SA-HRP and anti-GFP antibodies to analyze the activity and expression level of PsbS-TurboID-eGFP. Each sample is a pool of ~ 20 seedlings (8mg). CBB: Coomassie Brilliant Blue; Molecular weight markers in kDa are shown on the left side of each figure.

Biotinylation activity of TurboID was examined in 5-day old selected independent PsbS transgenic lines (PsbS-TurboID-eGFP lines #17, #18, and #21) (**Figure 3A**) and the control transgenic lines (PTP-TurboID-eGFP, lines #15, #19, and #20) (**Figure 3B**), respectively. TurboID showed significantly higher activity in both the selected PsbS-TurboID-eGFP transgenic lines and the control (PTP-TurboID-eGFP) transgenic lines within 1 h after biotin treatment in comparison with that in untransformed WT seedlings (**Figures 3A, B**). These results suggest that both PsbS-TurboID-eGFP and PTP-TurboID-eGFP constructs are functional and are capable of catalyzing PL efficiently in the transgenic plants when submerged in the biotin solution.

Optimizing the experimental conditions for biotinylation is crucial for identifying potential interacting factors and the proximal proteome of the target proteins (Mair et al., 2019). We therefore investigated the optimal experimental conditions for PL with the PsbS-TurboID-eGFP fusion protein in the selected Arabidopsis transgenic plants (**Figures 3C, D**), by comparing them with the optimal experimental conditions for biotinylation of TurboID in the control TurboID-eGFP transgenic plants (**Supplementary Figure S3**).

In TurboID-eGFP seedlings, the activity of TurboID, for which biotinylated proteins were visualized by immunoblot, was nearly as high at 22°C as at 30°C and 37°C (**Supplementary Figure S3A**), indicating TurboID-mediated biotin labeling can be performed at room temperature. To determine optimal substrate concentration for TurboID, biotin concentrations ranging from 1 to 250 µM were tested in both TurboID-eGFP and PsbS-TurboID-eGFP seedlings by comparison with that in untransformed WT seedlings. The results showed that only weak background labeling occurred at 10 µM biotin, then increased sharply at 50 µM, and reached saturation at 100 µM in TurboID-eGFP (**Supplementary Figure S3B**) and a similar biotin labeling pattern was also observed in PsbS-TurboID-eGFP seedlings (**Figure 3C**). These results suggest that a biotin concentration of 50 µM is optimal for TurboID-mediated biotin labeling in the transgenic plants.

We further determined the appropriate labeling time for TurboID-mediated protein biotinylation. TurboID in both PsbS-TurboID-eGFP and TurboID-eGFP samples could induce protein labeling above background levels within 30 minutes of treatment with 50 µM biotin at room temperature (22°C), and the labeling increased sharply and reached saturation after 60 min (**Figure 3D**; **Supplementary Figure S3C**). In addition, a 3-hour incubation time could increase the amount of detected protein without a new protein band appearing (**Figure 3D**; **Supplementary Figure S3C**). Thus, the biotin PL in the following study was performed under 50 µM biotin conditions for 3h at room temperature.

### 3.3 Localization and function of PsbS-TurboID-eGFP after stable expression in *Arabidopsis*

**Figure 4:**
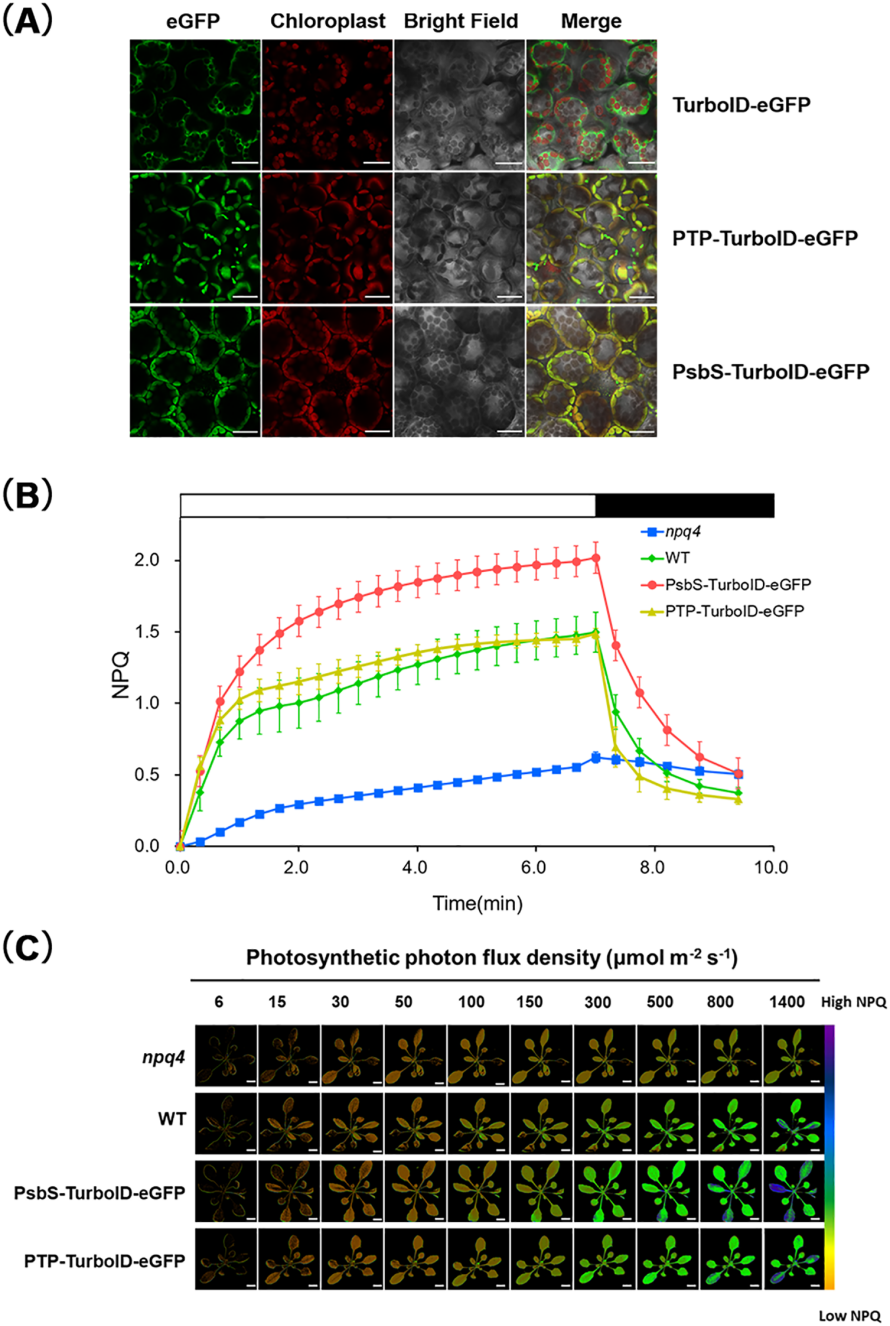
Subcellular localization and NPQ measurement of PsbS-TurboID-eGFP. **(A)** Laser confocal microscopy images of mesophyll cells of 5-day old transgenic seedlings stably expressing PsbS-TurboID-eGFP (#18), PTP-TurboID-eGFP(#19) and TurboID-eGFP(#3), respectively. Seedlings were grown under the indicated culture condition. eGFP (green) and the autofluorescence of chlorophyll (red). Scale bar = 50 μm. **(B)** The induction of non-photochemical quenching (NPQ) in leaves of wild-type (WT, Col-0 ecotype) and *npq4*(PsbS-defective mutant), transgenic plants PsbS-TurboID-eGFP (#18) and PTP-TurboID-eGFP(#19) under the high light (HL, 685μmol·m^−^²·s^−^¹) condition. After a 2-hour dark treatment, 2-week-old seedling were treated with HL for 7 minutes (white box), followed by a 2-minute and 24-second dark (black box) to relax qE. Data are presented as mean ± S.D (n=6). **(C)** The rapid NPQ determination of *npq4*, WT, PsbS-TurboID-eGFP (#18) and PTP-TurboID-eGFP(#19) transgenic plants. Briefly, after 2 hours of dark adaptation, the plants were treated with the light intensity increased every 30 seconds in sequence as indicated. Images of maximum chlorophyll fluorescence (Fm and Fm’) were obtained before actinic light illumination and within 30 s of illumination, and then digitally processed to generate a NPQ false-color image of (Fm - Fm’)/Fm’. The NPQ image is represented by a “rainbow” range from red to purple. Scale bar = 1 cm.

As mentioned above, PsbS is a nuclear-encoded nascent protein, which is targeted to the thylakoid membrane from the cytosol after translation through multiple complex steps, including cytosolic sorting, translocation across the envelope membranes, sorting in the stroma and insertion into the thylakoid membrane (Cline and Dabney-Smith, 2008). To confirm proper localization of PsbS-TurboID-eGFP expression, the GFP signal in subcellular compartments of the transgenic plant leaves was examined. We observed the GFP signal throughout the cytoplasm, but not in the chloroplasts in mesophyll cells of TurboID-eGFP plants. In contrast, the GFP signal in cellular compartments of PTP-TurboID-eGFP and PsbS-TurboID-eGFP was observed to predominantly overlap with chloroplast autofluorescence but was not present in the cytosol (**Figure 4A**), indicating that both PTP-TurboID-eGFP and PsbS-TurboID-eGFP fusion proteins were imported into chloroplasts.

PsbS was reported to function as a photoprotective “switch” in the light-harvesting antenna in the thylakoid membrane of chloroplast (Correa-Galvis et al., 2016). In order to further confirm the function of PsbS-TurboID-eGFP, we determined the NPQ in PsbS-TurboID-eGFP plants in comparison with the controls PTP-TurboID-eGFP, WT and *npq4*. The NPQ in PsbS-TurboID-eGFP was approximately 140% higher than the WT level, while NPQ in *npq4* was reduced to about 70% of the WT level, suggesting the function of the transgene PsbS-TurboID-eGFP during NPQ under the HL condition. In addition, the NPQ of PTP-TurboID-eGFP was no significantly different from that of WT (**Figures 4B, C**), indicating it could be used as a control for PsbS-TurboID-eGFP in subsequent PL experiments.

### 3.4 Identification of the PsbS interactome under dark and high light conditions

To capture the dynamic, highly transient protein interactions of PsbS in the thylakoid membrane during NPQ, we employed *in situ* PL (TurboID) to investigate its interactome in Arabidopsis seedlings stably expressing PsbS-TurboID-eGFP under the HL (600 µmol·m^−^²·s^−^¹) condition (named PsbS-HL) compared to control seedlings expressing PTP-TurboID-eGFP under the HL condition (PTP-HL) (**Figures 2**-**4**). PsbS-HL and PTP-HL seedlings were incubated with 50 μM biotin for 3 h at 22 °C. This condition maintained optimal TurboID activity in both lines (**Figure 3**), while inducing significantly higher NPQ levels in PsbS-HL seedlings compared to those of PTP-HL and WT controls (**Figures 4B, C**). PsbS-TurboID-eGFP seedlings under the dark condition (PsbS-D) and PTP-TurboID-eGFP seedlings under the dark (PTP-D) served as controls to identify light-responsive PsbS interaction partners.

Large-scale streptavidin pull-downs followed by mass spectrometry analysis were performed on four sample groups: PsbS-HL, PTP-HL, PsbS-D, and PTP-D (**Figures 1A-C**; **Supplementary Figure S4**). Stringent filtering retained only proteins consistently identified across three biological replicates for further analysis. Comparative analysis showed that in Arabidopsis plants expressing PsbS-TurboID-eGFP, 1,322 biotinylated proteins were identified under dark conditions (PsbS-D), and 1,179 biotinylated proteins were identified under high light conditions (PsbS-HL). In contrast, the control plants expressing PTP-TurboID-eGFP had 504 biotinylated proteins under dark conditions (PTP-D) and 803 biotinylated proteins under high light conditions (PTP-HL) (**Figure 1D**; **Supplementary Table S1**). **Figure 1E** showed that 1,322 proteins in the PsbS-D and 1,179 proteins in the PsbS-HL samples were identified. Among these identified proteins, 1,086 proteins (76.8% of total 1,415 proteins) were overlap. In addition, principal component analysis (PCA) also showed PsbS-D and PsbS-HL samples clustered together, while distinct separation of PTP samples under dark versus HL conditions. This may be attributed to light-induced physiological changes in chloroplasts altering non-specific biotinylation by TurboID, leading to separation of PTP samples under different light conditions, despite the transit peptide lacking PsbS activity. These results suggest that there is substantial overlap in the PsbS-HL and PsbS-D interactomes (**Figure 1F**).

Gene Ontology (GO) enrichment analysis of these PsbS-associated proteins in the PsbS-HL and PsbS-D samples identified conserved processes across, including photosynthesis, chloroplast/thylakoid membrane organization, chlorophyll biosynthesis, translation, cold response, and the reductive pentose-phosphate cycle (**Figures 1G, H**). Subcellular localization prediction revealed that 63% of PsbS-interacting proteins were chloroplast-localized, with 16% specifically associated with the thylakoid membrane (**Figures 1G, H**; **Supplementary Table S2**).

**Figure 5:**
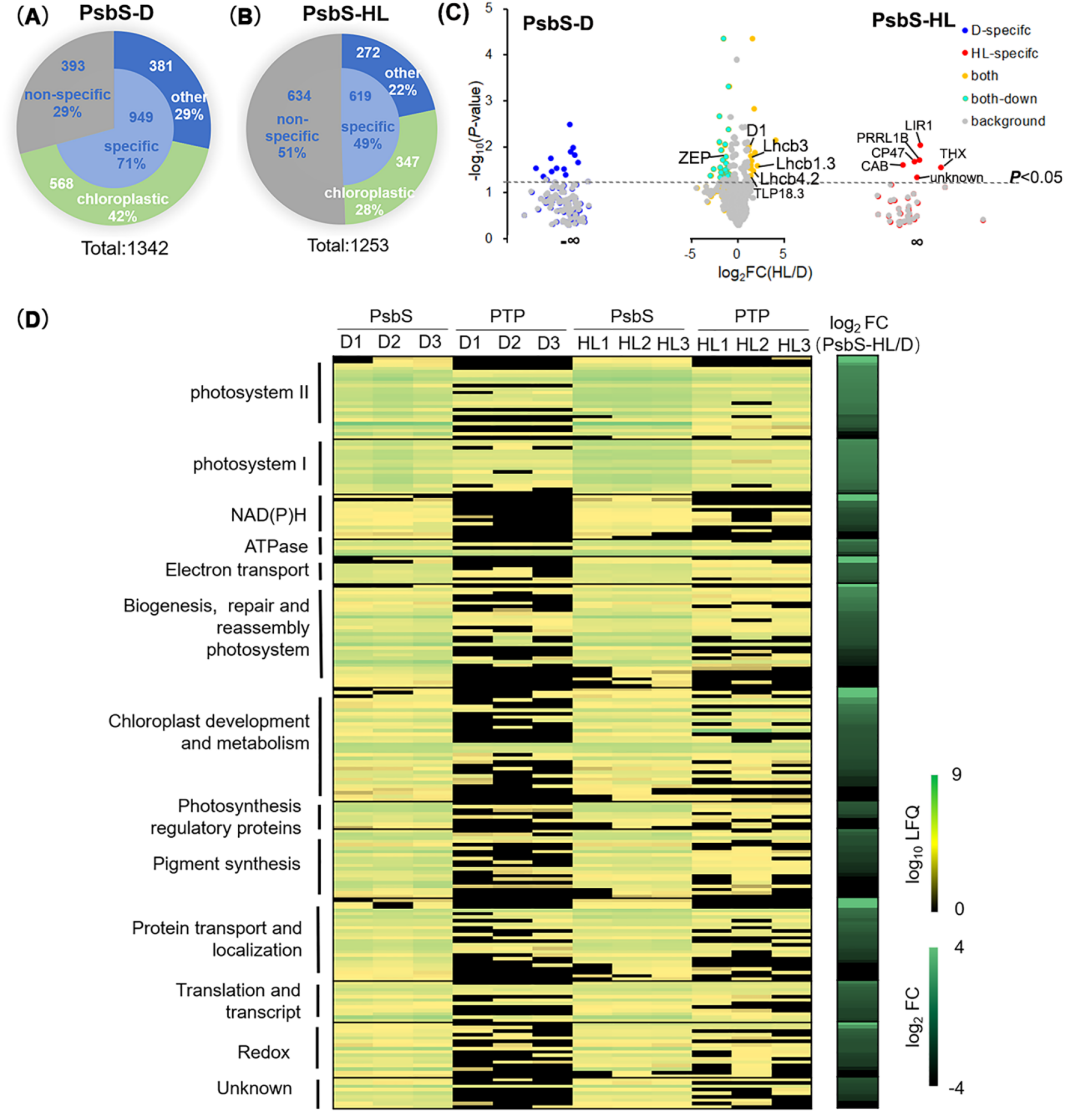
Chloroplast-specific PsbS proximity-labeling group and PsbS-binding proteins in thylakoid. **(A, B)** Pie charts illustrating the quantification of specific and non-specific binding proteins identified in the PsbS proximity-labeling proteome under dark **(A)** and high light **(B)** conditions. Specific binding proteins are further categorized into chloroplast-localized proteins and proteins localized to other cellular compartments. **(C)** Scatter plot depicting the relative differences (-log_10_*P*-value, y-axis) in chloroplast protein abundance changes (log_2_fold change (FC), x-axis) between plants expressing PsbS-TurboID-eGFP under dark **(D)** and high light (HL) conditions. **(D)** Heatmap representing the cluster analysis of PsbS-specific binding proteins in the thylakoid membrane. The left panel displays the log_10_ label-free quantification (LFQ) intensity for PsbS-D, PTP-D, PsbS-HL, and PTP-HL. Data from three independent biological replicates are denoted as D1, D2, D3 (dark conditions) and HL1, HL2, HL3 (high light conditions). The right panel illustrates the log_2_ fold-change (log_2_FC) of PsbS-HL and PsbS-D proteins.

To characterize the categories of biotinylated proteins specifically binding to PsbS, proteins were categorized based on enrichment value comparison. Proteins with log_2_(PsbS LFQ intensity/PTP LFQ intensity) <1 were classified as “non-specific” binding proteins, while those showing significant abundance in PsbS versus PTP (log_2_ FC ≥1, *P*-value<0.05) under the dark or the HL conditions were designated as “specific” binding proteins. Proteins exhibiting reduced abundance in PsbS-D and PsbS-HL compared to controls PTP-D or PTP-HL, respectively, were excluded and thus classified as “non-specific”. Excluding 393 and 634 ‘non-specific’ proteins in the dark and the HL condition, respectively (**Figures 5A, B**; **Supplementary Table S3**), we identified 949 and 619 PsbS-specific binding proteins in the PsbS-D and the PsbS-HL samples, respectively. Of these, 568 proteins in the PsbS-D samples and 347 proteins in the PsbS-HL sample were exclusively localized in chloroplasts (**Figures 5A-C**; **Supplementary Table S3**). Among these chloroplastic proteins, 218 high-confidence PsbS-proximal proteins localized to thylakoid membranes (**Figure 5D**; **Supplementary Figures S5A, B**). Cross-referencing with PsbS interaction proteins using immunoaffinity/immunoprecipitation approaches in previous studies (Fantuzzi et al., 2023; Correa-Galvis et al., 2016; Teardo et al., 2007) revealed 43 identifiable proteins (20%) among the 218 PsbS-proximal proteins in this study overlapped with published datasets (**Supplementary Table S4**). Notably, 175 proteins (80%) identified in this study represent novel PsbS-associated candidates.

DAVID functional enrichment analysis revealed these 218 PsbS-proximal proteins in thylakoid membranes were exclusively associated with photosynthetic pathways and related thylakoid processes: biogenesis/repair/reassembly of photosystems (30 proteins, 14% of total 218 proteins), PSII (24 proteins, 11%), PSI (16 proteins, 7%), redox regulation (16 proteins, 7%), NAD(P)H complex (13 proteins, 6%), chloroplast development/metabolism (33 proteins, 15%), cytochrome b_6_f complex/electron transport (8 proteins, 4%), photosynthetic regulation (8 proteins, 4%), protein transport/localization (24 proteins, 11%), pigment synthesis (20 proteins, 9%), translation/transcription (12 proteins, 6%), ATPase complex (5 proteins, 2%), and functionally uncharacterized proteins (9 proteins, 4%) (**Figure 5D**; **Supplementary Figure S5C**). These findings suggest that PsbS interacts with multiple proteins within thylakoid membranes.

**Figure 6:**
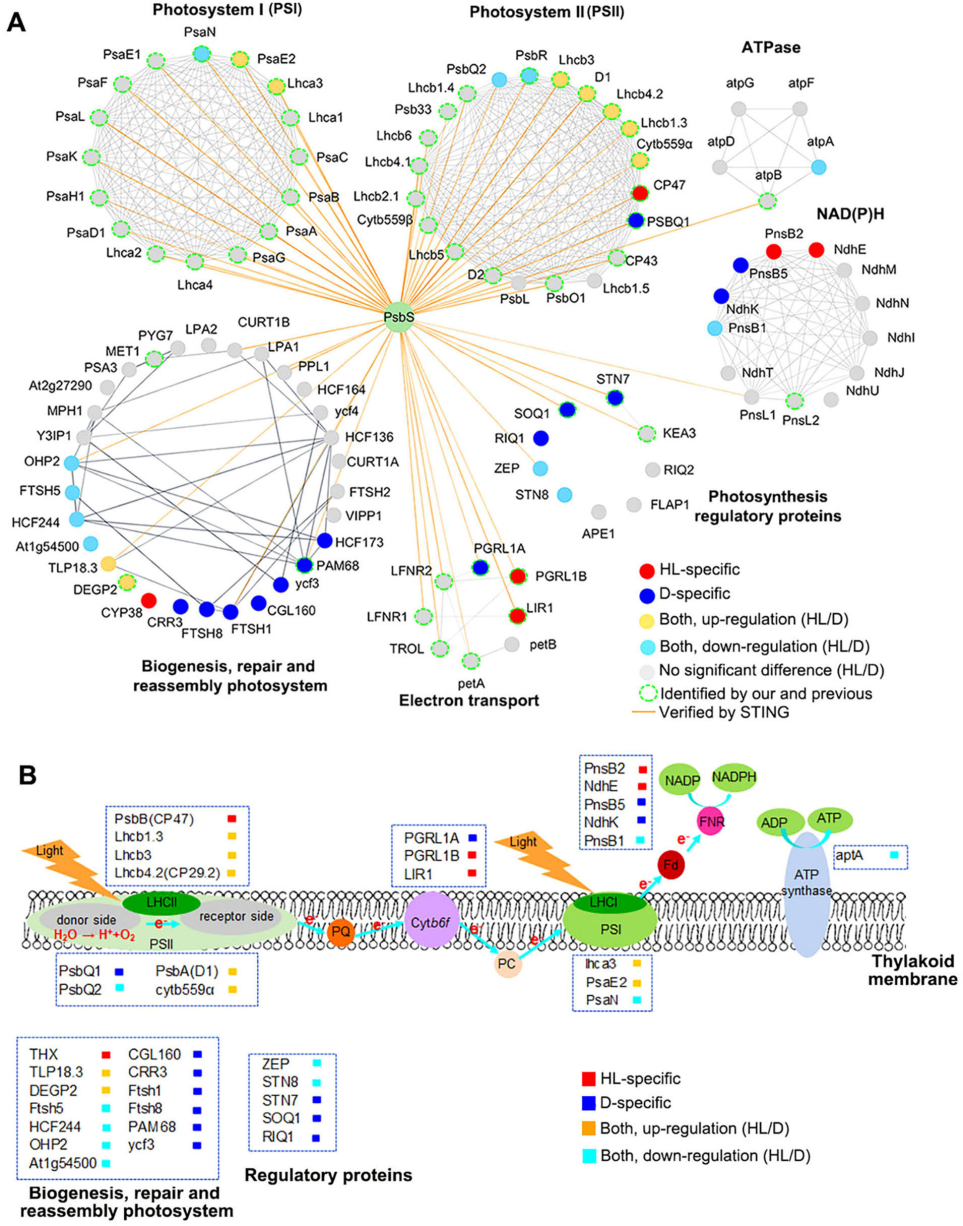
The interaction network of PsbS proximity proteins and possible functional characterization. **(A)** Graphical representation of the hypothetical protein-protein interactions. All nodes represent photosynthetic-related interaction partners of PsbS. Lines represent interactions from the STRING database with confidence ≥ 0.7. Orange lines represent interactions with PsbS are verified by the STRING database. Each module in the network is named using GENEONTOLOGY for gene ontology analysis. **(B)** proposed model for possible interactions of PsbS with the subunits from different large protein complexes in the thylakoid membrane.

By combining our data with PsbS interaction data from public databases using chemical crosslinking, immunolabeling and magnetic-bead-linked antibody pull-down (Correa-Galvis et al., 2016; Sacharz et al., 2017; Teardo et al., 2007), we uncovered distinct cluster corresponding to putative PsbS- neighboring proteome under the D and the HL conditions, identifying as three different patterns: type 1 is composed of high light (HL)-specific PsbS binding proteins, which are particularly interested, as they might be more directly related to the role of PsbS in NPQ regulation. These proteins include PS II reaction center protein CP47, Light-regulated protein 1 (LIR1), thioredoxin X(THX), a function unknown chlorophyll a-b binding proteins (Q7M1L1, CAB), and proton gradient regulation 5-like photosynthetic phenotype 1B (PGRL1B) (**Figure 5C**). Type 2 include dark (D)-specific PsbS binding proteins, which could play roles involving in PsbS function in thylakoid membranes in the NPQ-inactive (dark), such as PSII protein PSBQ1, proteins functional relation to biogenesis, repair and reassembly photosystem (FtSH1, FtSH8, HCF173, etc.), three proteins involved in photosynthesis regulatory function (RIQ1, SOQ1 and STN7), two proteins in NAD (P)H (NdhK and PnsB5) and one in electron transport (PGRL1A). Type 3 proteins were identified binding to PsbS under both the HL and the D conditions, but with different abundance. This type proteins include multiple members of PSII proteins (D1, Lhcb1.3, Lhcb3 and Lhcb 4.2, etc.), photosynthesis regulatory proteins (STN8 and ZEP), and biogenesis, repair and reassembly photosystem (TLP18.3 and DEGP2) (**Figures 5C, D**; **Supplementary Table S4**). Among these components, Lhcb1.3 showed the most obvious enrichment (3.98-fold) and the TLP18.3 protein, which are crucial for the assembly and repair of the PSII supercomplex, was up-regulated (about 2.5-fold) in PsbS-HL compared to PsbS-D, while zeaxanthin epoxidase (ZEP) is a TurboID-labeled component that preferentially binds under dark conditions (decreased 2.4-fold) in the PsbS-HL compared PsbS-D (**Figure 5C**; **Supplementary Table S4**). Furthermore, STRING protein-protein interaction (PPI) network (minimum edge score≥0.7, v12.0) base on above three types of PsbS-proximal proteins were constructed to summarize PsbS interactome in the network (**Figure 6**, **Supplementary Table S4**).

### 3.5 Functional verification of the specific binding proteins of PsbS under high light conditions

**Figure 7:**
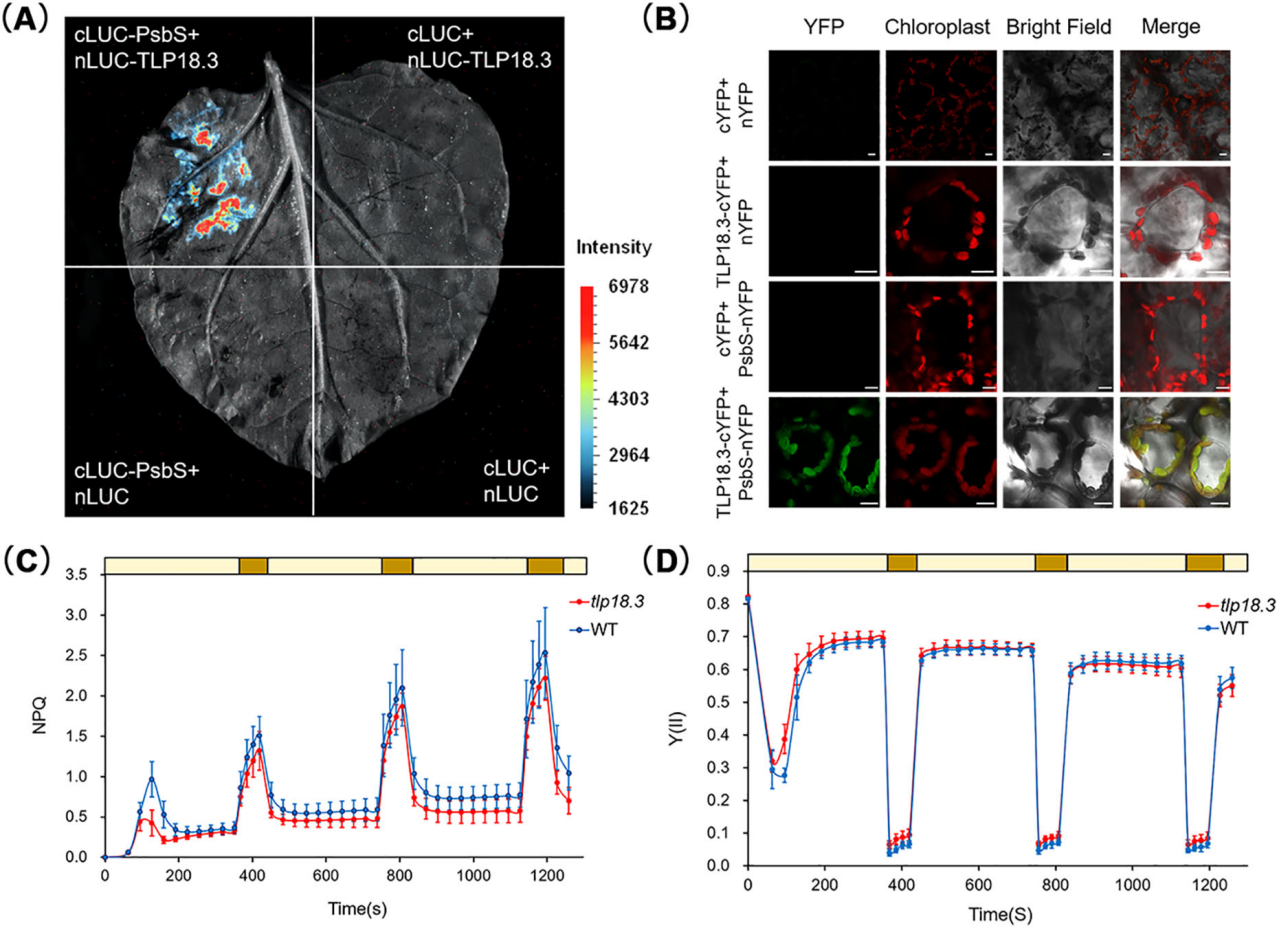
Verification of the interaction between PsbS and TLP18.3. **(A)** The luciferase (LUC) complementation assay was used to verify the interactions between PsbS and TLP18.3 in *N. benthamiana* intact leaves. A vector in which the C-terminal fragment of luciferase was fused between the transit peptide and the functional fragment of the PsbS protein (cLUC-PsbS) was constructed. This vector was co-transformed with the vector of the N-terminal fragment of luciferase fused to TLP18.3 (nLUC-TLP18.3), as shown in the upper left corner. cLUC-PsbS with empty nLUC, nLUC-TLP18.3 with empty cLUC, and empty cLUC with empty nLUC were set as negative controls to exclude non-specific interactions. Scale bar = 1 cm. **(B)** The bimolecular fluorescence complementation assay (BiFC) was used to verify the interaction between PsbS and TLP18.3. A recombinant vector in which the N-terminal fragment of yellow fluorescent protein was fused between the transit peptide and the functional fragment of PsbS (PsbS-nYFP) and a vector in which TLP18.3 was fused to the C-terminal fragment of yellow fluorescent protein (cYFP-TLP18.3) were constructed respectively. These two vectors were co-transformed into tobacco leaves via the Agrobacterium-mediated method. At the same time, PsbS-nYFP with empty cYFP, cYFP-TLP18.3 with empty nYFP, and empty cYFP with empty nYFP were set as negative controls to accurately determine whether the experimental results were due to specific interactions. When observed under a laser confocal microscope, the autofluorescence of chlorophyll represents the chloroplast. Scale bar = 10 μm **(C, D)** Measurement of NPQ and Y(II) of WT and *tlp18.3* under fluctuating light was performed. After growing on MS medium for 1 week, WT and *tlp18.3* control seedlings of the same age were transferred to soil and cultured under long-day conditions for 2 weeks. After a 2-hour dark treatment, the light intensity for high light treatment was 552 μmol·m^−^²·s^−^¹ (dark yellow), and then the light intensity for low light treatment was 42 μmol·m^−^²·s^−^¹ (light yellow). Data are presented as mean ± SD (n = 6).

**Figure 8:**
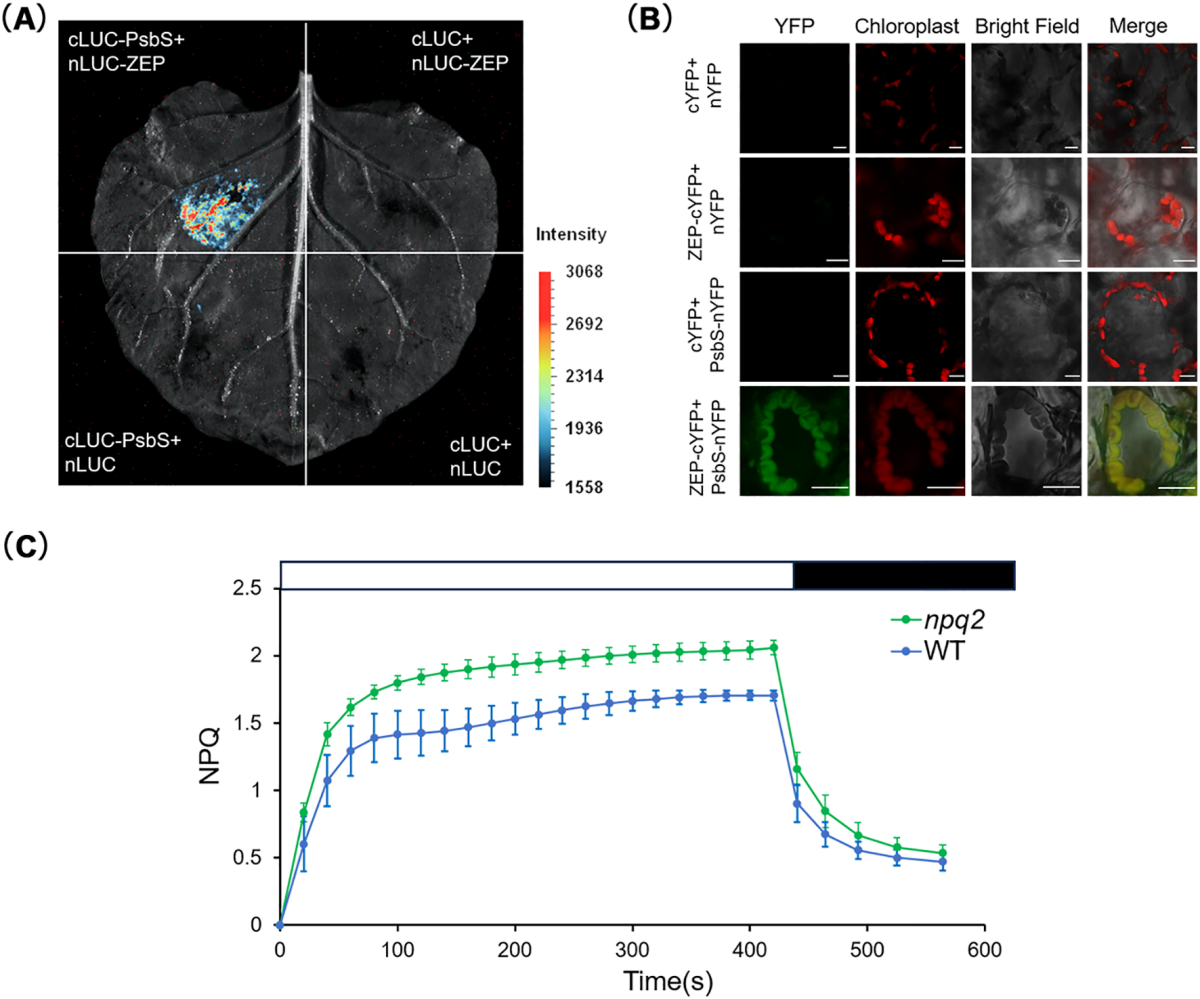
Verification of the interaction between PsbS and ZEP. **(A)** The luciferase complementation assay was used to verify the interactions between PsbS and ZEP. The cLUC-PsbS and nLUC-ZEP encoding ZEP were co-transformed in *N. benthamiana* intact leaves (upper left corner). Corresponding negative controls were set as described in **Figure 7A** to ensure the reliability of experimental results. Scale bar = 1 cm. **(B)** The bimolecular fluorescence complementation assay (BiFC) was used to verify the interactions between PsbS and ZEP. The vector encoding PsbS-nYFP and the vector encoding ZEP fused to cYFP (cYFP-ZEP), together with corresponding negative controls, were co-transformed into tobacco leaves using the Agrobacterium-mediated method as described in **Figure 7B**. Scale bar = 10 μm. **(C)** Measurement of NPQ induced by a time-course at 685 μmol·m^−^²·s^−^¹ was performed in WT and *npq2*(ZEP-defective mutant). The experimental materials were the same as above. After 2 hours of dark treatment, light treatment was carried out for 7 minutes (white box), and then dark treatment was carried out for 2 minutes and 24 seconds (black box) to relax qE. Data are presented as mean ± S.D. (n = 6).

To validate the proximity labeling PsbS binding proteome data, we examined the interactions of PsbS between two selected candidate binding proteins, TLP18.3 (AT1G54780, thylakoid lumen protein of 18.3kDa), and ZEP (AT5G67030, Zeaxanthin epoxidase), respectively, using the Luciferase Complementation Assay (LCA) and Bimolecular Fluorescence Complementation (BiFC) assay. By co-transforming tobacco leaves with cLUC-PsbS and nLUC-TLP18.3/nLUC-ZEP, significant luciferase activity was observed (**Figures 7A** and **8A**). The BiFC assay further showed that yellow fluorescent protein expression occurred in mesophyll cells of tobacco leaves co-expressing PsbS-nYFP and cYFP-TLP18.3 and cYFP-ZEP, respectively (**Figures 7B** and **8B**), confirming the interaction with PsbS and these two selected candidate proteins.

To further examine the function of interaction of PsbS with TLP18.3, the NPQ and PSII actual photochemical efficiency (Y(II)) in the *tlp18.3* (Salk_050942) plants, was compared with those in the WT plants under the same conditions (**Figures 7C, D**; **Supplementary Figures S6A, B**). The results showed that there were no significant differences in NPQ and Y(II) between the *tlp18.3* mutant and the WT under steady-state HL conditions (**Figures 6A, B**). Previous studies have reported that the *tlp18.3* mutant is more sensitive to PSII photoinhibition and shows faster declines in photochemical efficiency under HL conditions (Jarvi et al., 2016), so we further measured NPQ and Y(II) in the *tlp18.3* mutant under fluctuating light conditions. The *tlp18.3* mutant under fluctuating light exhibited significantly reduced NPQ capacity and abnormal Y(II) responses (**Figures 7C, D**). For example, the NPQ of the *tlp18.3* was significantly lower than that of the WT, while the Y(II) was slightly lower than that of the WT during low-light period of the third fluctuating light cycle (**Figures 7C, D**). Different from its responses to the fluctuating light conditions, the *tlp18.3* mutant grown under the HL conditions exhibited lower maximum NPQ and higher maximum Y(II) compared to the WT. These results indicate that the *tlp18.3* mutant is more sensitive to fluctuating light conditions. To further test the function of the other potential PsbS binding protein ZEP, we also measured NPQ in the ZEP mutant *npq2* (Salk_027326C) and found that NPQ in the *npq2* mutant was significantly higher than that in the WT under the HL conditions (**Figure 8C**).

## 4 Discussion

### 4.1 TurboID-mediated PL as an effective method to identify interacting proteins of PsbS

In this study, we employed TurboID-mediated PL to systematically characterize the PsbS interactome in *Arabidopsis* seedlings under both dark and HL conditions. TurboID can support *in situ* protein labeling within physiological subcellular microenvironments of living cells (Branon et al., 2018), which can minimizes artifacts commonly associated with conventional pull-down and co-immunoprecipitation techniques (Zhang et al., 2020, 2022).

Using TurboID labeling, we find PsbS interactome network under high light. The high enrichment of biotinylated PsbS in PsbS-HL and PsbS-D samples compared to PTP controls indicates the specificity of TurboID-mediated PL, as the signal depends on functional PsbS rather than non-specific biotinylation. The PL results successfully recapitulated known PsbS interactors, such as the key NPQ-associated partners Lhcb1.3, Lhcb4.2, which have been verified by previous studies using chemical crosslinking and immunolabeling (Correa-Galvis et al., 2016), supporting the ability of this approach in identifying the interactome of PsbS *in vitro*.

### 4.2 The location of PsbS in thylakoid membrane: evidence supporting a widespread existence of PsbS in thylakoid membrane rather than staying inside light harvesting complexes

Great effort has been made to identify the location of PsbS in the thylakoid membrane (Correa-Galvis et al., 2016; Nield et al., 2000; Sacharz et al., 2017). For example, the analysis of C_2_S_2_M_2_-PSII-LHCII super complex components using cryo-electron microscopy didn’t find PsbS as an intrinsic part of the PSII-LHCII super complex, although a cleft in the complex was assumed as potential position of PsbS protein (Su et al., 2017). The PsbS binding partners were also examined by chemical crosslinking and immunolabelling and suggested that PsbS may be located around the PSII-LHCII supercomplex, while increasing interaction with LHCII in the HL conditions (Correa-Galvis et al., 2016). Although the dynamic localization of PsbS has not been fully elucidated, a notion that PsbS protein is distributed in multiple locations of both the grana regions and the stroma regions, or exists in different states, such as monomers or dimers, was proposed (Valencia and Pandit, 2024). However, the exact location of PsbS in the thylakoid remains unclear. In this current study, when TurboID and eGFP was attached to PsbS protein, this PsbS-TurboID-eGFP can still enhance the NPQ phenotype of WT (**Figure 4B**), suggesting that the PsbS-TurboID-eGFP played its typical function, either with the attachment of TurboID and GFP. Considering that the molecular weight of PsbS-TurboID-eGFP is approximately 90 kDa, which is greater than the dimension of the cleft in the C_2_S_2_M_2_-PSII-LHCII complex (Su et al., 2017; Valencia and Pandit, 2024), it is likely that PsbS does not reside in this cleft.

This new analysis not only validated previously reported interactions but also identified novel candidate PsbS-interacting proteins (**Figure 6**). Functional annotation clustering analysis using DAVID revealed that the most prominently enriched GO terms for biological processes and cellular components were photosynthesis-related activities and chloroplast localization, respectively (**Figures 1G, H**; **Supplementary Table S3**). The majority of these newly identified interactors were chloroplast-localized proteins, while some of the interactors were also predicted to localize to cytosol. The reason for PsbS to have interacting proteins in cytosol could be that PsbS is a nuclear-encoded protein, which undergoes cytosolic synthesis as a precursor before chloroplast import. Here we emphasize that some cytosolic-localized proteins together with a minor proportion of false-positive candidate proteins, such as Histone H4, were already excluded through a stringent identification criterion.

In addition to interactions with PSII-associated proteins we have described above, our investigation also revealed that PsbS also interacts with some components of PSI (**Figures 5D** and **6**). This observation is consistent with previous findings, who show that some PSI proteins can interact with PsbS (Teardo et al., 2007; Correa-Galvis et al., 2016). We have discovered that the PsbS binds protein assembly factors YCF3, YCF4 and Y3IP1, which play a role in the formation of PSI (Albus et al., 2010; Nellaepalli et al., 2018). Notably, we also found the interaction of PsbS with the components of monomeric minor antenna proteins CP29/CP24 and the trimeric LHCII complex, which were demonstrated to regulate the dynamic assembly-disassembly equilibrium of a PSII-LHCII supercomplex by binding (Betterle et al., 2009; Guardini et al., 2020; Dall’Osto et al., 2017). Most cyclic electron transport proteins interacting with PsbS were down-regulated, which is consistent with previous studies that the level of PsbS in plants has a regulatory role in cyclic electron transport (Roach and Krieger-Liszkay, 2012). In addition, some critical PSII auxiliary factors, such as TLP18.3, FTSH2 and DegP2 (Sirpio et al., 2007; Kanervo et al., 2003; Dogra et al., 2017), were also identified as PsbS-binding partners in this study (**Figure 5**). These results on one hand can be interpreted as that PsbS binding partners could be in multiple locations in the thylakoid membrane, on the other hand, it might also suggest that PsbS might stay in the interface between these different super-complexes, i.e. PsbS might stay in the interface between LHCII, LHCI, and PSI, instead of staying in the cleft of LHCII as discussed earlier.

### 4.3 PsbS proximity proteins displayed three distinct patterns

Current models propose that PsbS mediates NPQ activation through dynamic, light-dependent interactions with PSII antenna proteins (Kiss et al., 2008; Nicol et al., 2019). Observations of light-enhanced PsbS-Lhcb1 trimer interactions within PSII complexes support this hypothesis (Correa-Galvis et al., 2016). PsbS retains NPQ modulation capacity in photosystem-depleted membranes from lincomycin-treated plants, suggesting its activity may not strictly require permanent association with specific binding partners in PSII reaction center (Ware et al., 2015), or in another word, the NPQ induction through PsbS only require functional light harvesting complex. Evidences also suggest that PsbS induction of NPQ is related to its ability to undergo significant conformational changes in response to lumen acidification under high light (Bergantino et al., 2003; Chiariello et al., 2023; Krishnan-Schmieden et al., 2021). During this period, PsbS was suggested to change from a dimeric state to a monomeric state (Li et al., 2004), which may cause substantial structural rearrangements of the photosynthetic antenna (Valencia and Pandit, 2024). However, the structural basis and HL-specific PsbS interaction partners remain unclear.

Our data show that PsbS interaction partners could display three distinct patterns: HL-specific (type 1), D-specific (type 2) and both HL- and D-proximity (type 3) (**Figures 5C** and **6**). Type 1 and Type 3 proteins might play an essential role in the ability of plant to respond rapidly to unpredictable changes in light intensity. We assumed that type 1 proteins, which are proximity to PsbS specifically under the HL, which might be more directly related to the role of PsbS in NPQ-active state regulation under the HL. The particular interaction of PsbS and CP47 under the HL is in agreement with previous analysis of Arabidopsis plants using chemical crosslinking (Correa-Galvis et al., 2016). In addition to a functional unknown chlorophyll a/b binding protein (Q7M1L1), some components related to electron transport (LIR1 and PGRL1B) and NAD(P)H complex (PnsB2 and NdhE), were also identified as type 1 proteins (**Figures 5C** and **6**). The reason for proximity of these proteins to PsbS under the HL remains unknown.

In contrast, type 3 proteins could bind to PsbS optimally in response to both the HL and the D conditions. In comparison with type 1 proteins, most of these proteins display moderate fold changes (2~5) increased binding abundance with PsbS under the HL or decrease abundance under the D condition. HL-induced increased interaction of PsbS with Lhcb1.3 (3.98-fold), Lhcb1.5 (2.31-fold), D1 (2.09-fold), CP43 (2.38-fold), Cyt b559α (2.21-fold), Lhcb3 (2.54-fold) and Lhcb4.2 (2.48-fold) compared to dark conditions (**Figures 5C, D**). Among these type 3 proteins, Lhcb1.3 and Lhcb4.2, which have been considered as directly binding to PsbS to mediate NPQ (Daskalakis, 2018; Correa-Galvis et al., 2016), have apparently increase in binding abundance in the HL condition. This result indicated that LHCII members (Lhcb1.3, Lhcb4.2, possible Lhcb 3), and RC members (CP47, D1 and cyb559α) in type 1 and type 3 proteins might act as primary interaction partners of PsbS during NPQ.

STRING protein-protein interaction (PPI) network of our data showed the proximity proteins of PsbS included subunits from all large protein complexes (PSII, PSI, cytochrome b6/f and ATP synthase) in the thylakoid membrane, but about 70% of them are associated with PSII (**Figure 6B**). In particularly, we also found many PsbS interaction partners from complexes of PSII auxiliary factors involved in biogenesis, repair and reassembly photosystem and regulatory proteins under the HL and the dark condition, respectively, suggesting that PsbS might not only interact with LHCII to produce quenched states during NPQ, but may also be involved in recovery PSII function by associating with auxiliary factors.

### 4.4 Co-regulation of NPQ and the repair process after photodamage

In our PL experiment, three critical thylakoid membrane auxiliary proteins involved in antioxidative defense and the repair of PSII, an uncharacterized thioredoxin (THX), TLP18.3 and DEP2, were also specially enriched in the PsbS-associated partners under the HL (**Figure 6**). Thioredoxin may be involved in a variety of redox reactions (Yokochi et al., 2021) and was assumed to play roles in maintaining the efficiency of light harvesting. For example, thioredoxin can inactivate STN7 kinase under HL conditions (Wu et al., 2021). *soq1*, a mutant lack a thylakoid-bound thioredoxin like protein was observed drastically enhanced NPQ at lower light intensities (Brooks et al., 2013), while dithiothreitol treatment decreased NPQ yield in Arabidopsis leaves (Naranjo et al., 2016). These previous studies indicated possible role of thioredoxins in regulating NPQ yield under high light. Our data further reveal PsbS is proximity to an uncharacterized THX specifically under the HL, suggesting the possibility of co-localization of PsbS and THX in thylakoid membrane, where NPQ yield under the HL condition. Currently, we couldn’t confirm the function of this uncharacterized THX in NPQ due to THX superfamily with redundancy function in the chloroplast stroma (Nikkanen and Rintamäki, 2019). Mutation of THX isoforms could be needed to further disclose new relationships between PsbS and THX during NPQ.

TLP18.3 was identified as a PsbS binding protein in response to the HL using PL in this study for the first time and the direct interaction between PsbS and TLP18.3 protein was also confirmed by luciferase complementation assay and BiFC (**Figures 7A, B**). TLP18.3 is a thylakoid lumen protein that plays a role in regulating the repair cycle of PSII in rapid response to fluctuating light and it has acid phosphatase activity (Wu et al., 2011; Sirpio et al., 2007). Interestingly, the tlp18.3 mutant show altered NPQ under fluctuating light, suggesting that TLP18.3 might be another factor involved in NPQ regulation as well (**Figures 7C, D**) (Jarvi et al., 2016). Whether this is a role of altered photosystems II due to the changed repair process or a direct role still needs to be studied. In either case, the direct involvement of TLP18.3 in both the repair process of PSII and also NPQ suggests a possibility of concurrent regulation of photoprotection and also photodamage and repair under HL.

Intriguingly, PsbS was found to interact with ZEP, which plays a major role in the xanthophyll cycle by regulating zeaxanthin content to influence NPQ (Kuster et al., 2023). Though ZEP is recognized as a critical enzyme involved in the xanthophyll cycle, however, how ZEP is involved in epoxidation of Zeaxanthin still needs to be elucidated.

The reconversion of zeaxanthin to violaxanthin is catalyzed by the ZEP which is localized in the chloroplast stroma (Schwarz et al., 2015). VDE is located in the thylakoid lumen, ​​whose activity is strictly modulated by the luminal pH (Hager and Holocher, 1994)​​. It becomes activated ​​when pH drops below 6.2, thereby ensuring catalysis of zeaxanthin synthesis ​​only when photosynthetic electron transport reaches light saturation (Pfundel and Dilley, 1993; Kuster et al., 2023)​​. The direct linkage of PsbS with ZEP mainly under the dark condition suggests a possibility that PsbS might cause conformational changes in the thylakoid membrane, which facilitate the transfer of zeaxanthin from lumen side to the stroma side for its epoxidation.

## 5 Conclusions

This study presents a systematic PL proteomic analysis of the PsbS binding proteins in response to both the dark and the HL condition is reported. This represent the first comprehensive catalog of the potential interacting partners of PsbS, which include both known partners and also novel partners, which can be used as a basis for systematic study mechanisms underlying NPQ. Analysis of the proteins interacting with PsbS under dark and light suggests a widespread existence of PsbS within thylakoid membrane, possibly in the interface among different photosystem super-complexes. The identified drastically altered interaction of PsbS with ZEP and also TLP18.3 suggests that the NPQ and repair process after photodamage might be under co-regulation.

## Data Availability

The mass spectrometry proteomics raw data were uploaded to the ProteomeXchange (proteomecentral.proteomexchange.org) with accession number PXD069971. All other supporting experimental data are available from the corresponding author upon reasonable request.

## Supplementary material

Supplementary Figure 1 Identification of homozygous lines of the *npq4* mutant.

Supplementary Figure 2 RT-PCR identification of PsbS-TurboID-eGFP and control transgenic plants.

Supplementary Figure 3 Optimization of experimental conditions for TurboID-eGFP proximity labeling.

Supplementary Figure 4 Immunoblotting to detect biotinylated proteins enriched by magnetic beads.

Supplementary Figure 5 Proximity-labeled proteins associated with PsbS under dark and high light conditions.

Supplementary Figure 6 Determination of NPQ and Photosystem II (Y(II)) in the *tlp18.3* mutant.

Supplementary Table 1 Proximity-labeling proteome data of PTP-TurboID-eGFP and PsbS-TurboID-eGFP under dark and high light conditions.

Supplementary Table 2 Gene Ontology (GO) enrichment analysis of PsbS proximity-labeled proteins under dark and high light conditions.

Supplementary Table 3 Identification and categorization of PsbS-interacting proteins as specific or non-specific under dark and high light conditions.

Supplementary Table 4 Comparative analysis of abundance variations in thylakoid-associated PsbS binding proteins between high light and dark conditions.

Supplementary Table 5 The list of primers.
