# Supplementary material for: Identification of PsbS binding proteins in Arabidopsis thaliana leaf chloroplasts under high light using TurboID-based proximity labeling
Source: Front Plant Sci. 2026 Jan 20;16:1705804. doi: 10.3389/fpls.2025.1705804 (PMC12864446; doi:10.3389/fpls.2025.1705804)
Supplement: Supplementary Figure 1 — Identification of homozygous lines of the npq4 mutant. [file DataSheet1.pdf]

Figure S1

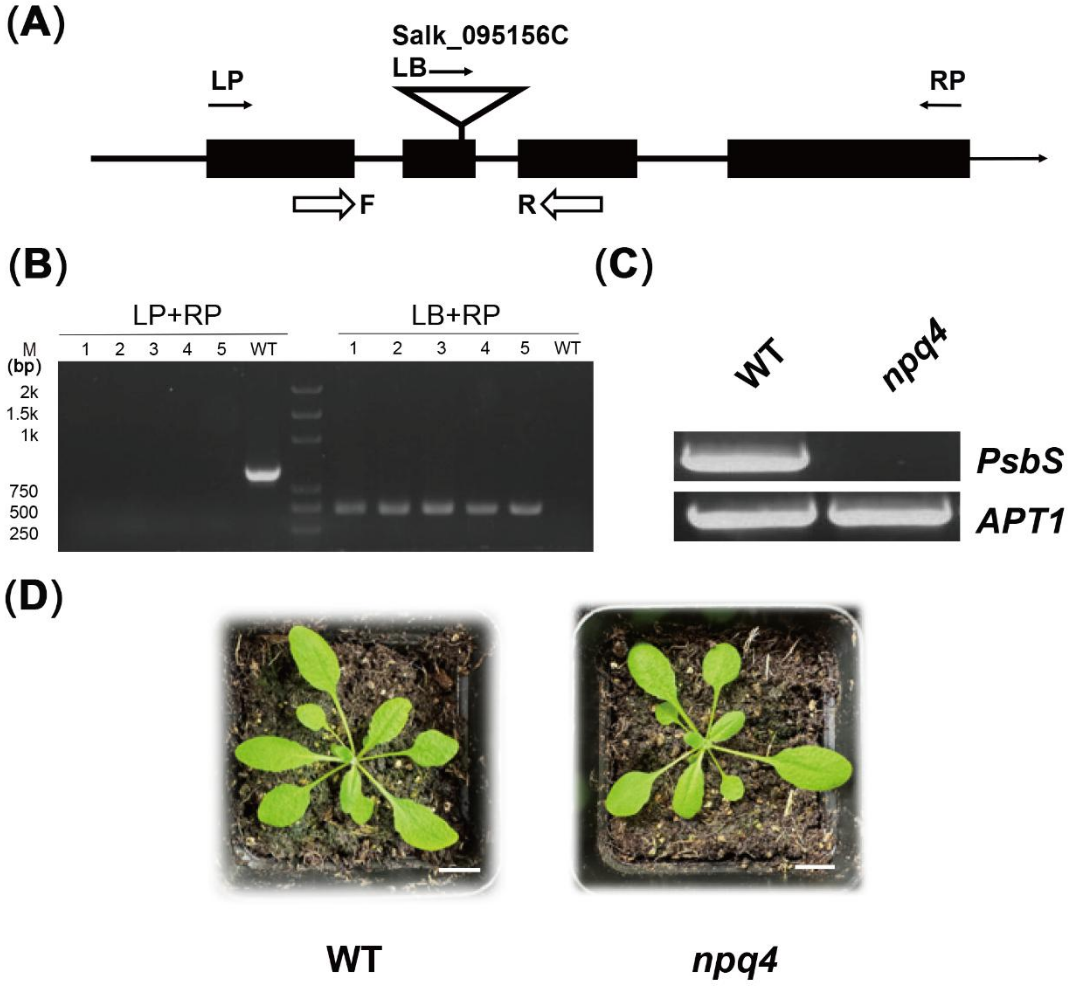

Figure S1 Identification of homozygous lines of the *npq4* mutant.

(A) Schematic diagram of the *PsbS* gene (AT1G44575) and localization of the T-DNA insertion in *npq4* (Salk_095156C). Exons and introns are represented by black boxes and lines, respectively. The white triangle indicates the T-DNA insertion site. Black arrows show the position and orientation of the primers used for genotyping and the white arrows for the position and orientation of the primers used for PsbS expression analysis by RT-PCR. (B) Validation of the *npq4* mutant lines by PCR analysis on genomic DNA. (C) RT-PCR verified *npq4* mutant at the transcriptional level. The APT1 gene (AT1G27450) was used as an internal reference for control. (D) Phenotypes of wild-type (WT) and *npq4* plants at three weeks after germination under long-day (16h light /8h dark) conditions at 22°C.

Figure S2

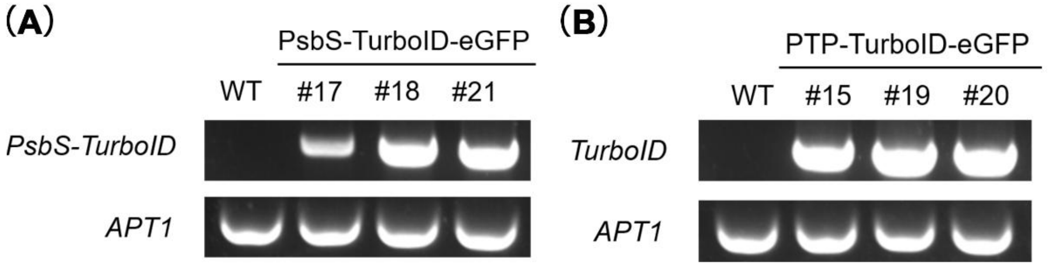

Figure S2 RT-PCR identification of PsbS-TurboID-eGFP and control transgenic plants. (A and B) Semi-quantitative RT-PCR was used to analyze the RNA expression levels of PsbS-TurboID in the leaves of transgenic PsbS-TurboID-eGFP plants (A) and TurboID in the leaves of PTP-TurboID-eGFP plants (B).

Figure S3

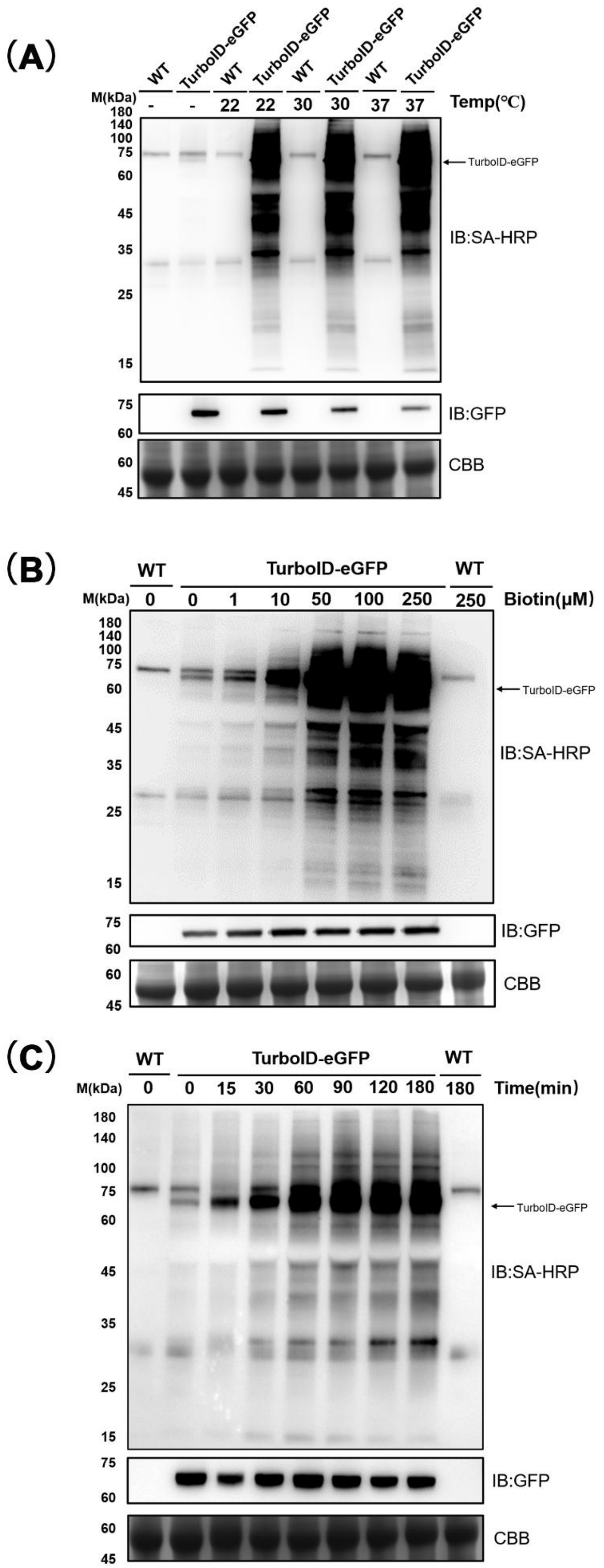
(A)
WT
TurboID-eGFP
M(kDa)
Temp(℃)
- - 22 22 30 30 37 37
180
140
100
75
60
45
35
25
15
TurboID-eGFP
IB:SA-HRP
IB:GFP
CBB
(B)
WT
TurboID-eGFP
WT
0 0 1 10 50 100 250 250
Biotin(μM)
IB:SA-HRP
IB:GFP
CBB
(C)
WT
TurboID-eGFP
WT
0 0 15 30 60 90 120 180 180
Time(min)
IB:SA-HRP
IB:GFP
CBB

Figure S3 Optimization of experimental conditions for TurboID-eGFP proximity labeling.

(A) WT and TurboID-eGFP(#3) transgenic plants were treated with a biotin solution at different temperatures for 5 hours. Immunoblotting was carried out using SA-HRP and anti-GFP antibodies to analyze the activity and expression level of TurboID-eGFP. (B) WT and TurboID-eGFP(#3) transgenic plants were treated with biotin solutions of different concentrations for 1 hour. Immunoblotting was performed using SA-HRP and anti-GFP antibodies to analyze the activity and expression level of TurboID-eGFP. (C) Wild-type and TurboID-eGFP(#3) Arabidopsis were treated with a 50 μM biotin solution for different durations. Then, proteins were extracted and immunoblot analysis was conducted to analyze the activity and expression level of TurboID-eGFP. SA-HRP: Streptavidin-Horseradish Peroxidase; CBB: Coomassie Brilliant Blue; Molecular weight markers (in kDa) are on the left side of each picture.

Figure S4

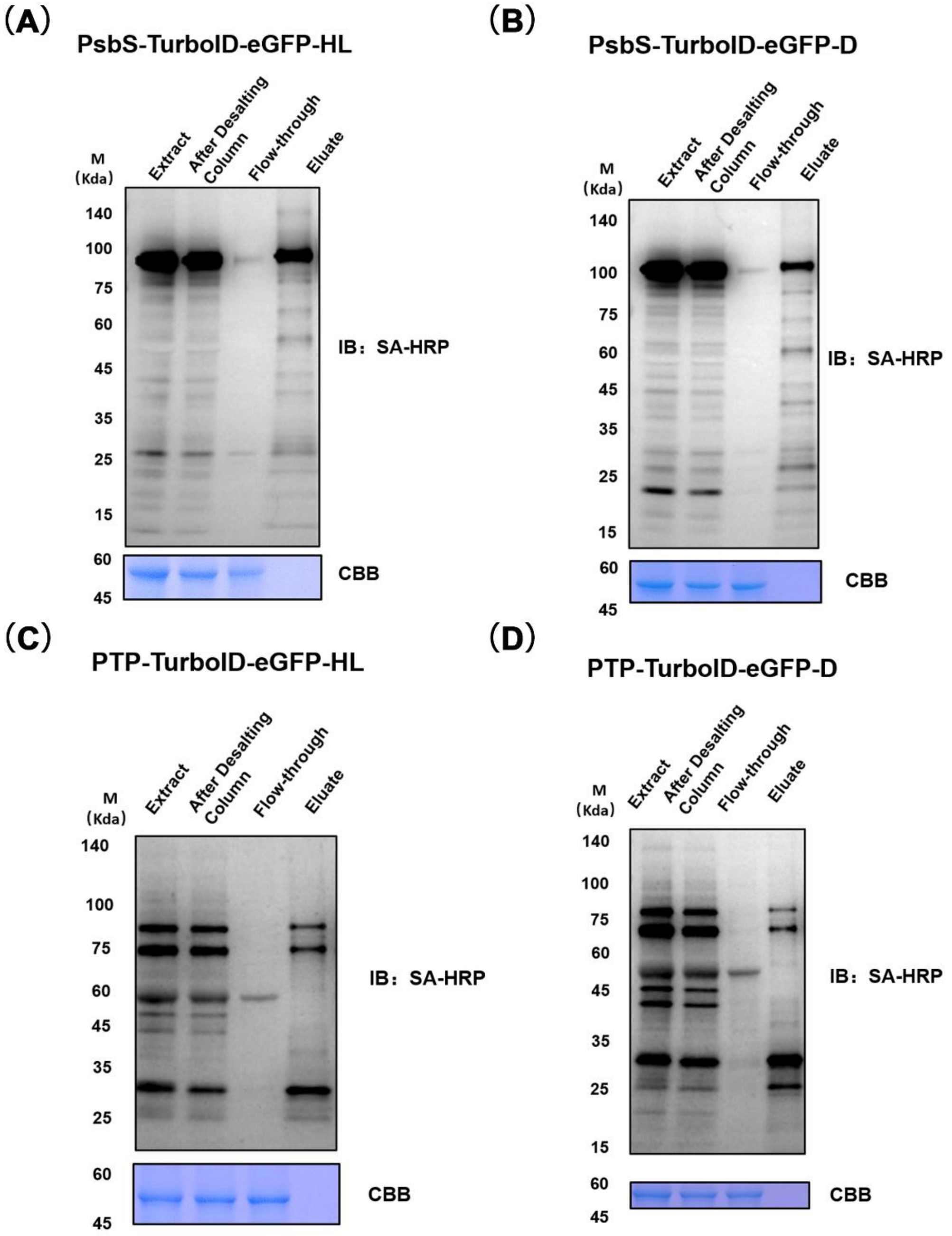

Figure S4 Immunoblotting to detect biotinylated proteins enriched by magnetic beads. (A and B) PsbS-TurboID-eGFP (#18) and (C and D) PTP-TurboID-eGFP (#19) *Arabidopsis thaliana* plants were treated with biotin (50 μM, 3 h) under high light (600 μmol $m^{-2}{\cdot}s^{-1}$, HL) and darkness (D), respectively. After protein extraction, the proteins were desalted using a desalting column. The desalted samples were incubated overnight with 120 μL of streptavidin magnetic beads, and finally eluted with 40 μL of 5× SDS Sample Buffer respectively. Extract: Protein supernatant after extraction and

centrifugation; After Desalting Column: Protein sample after desalting; Flow-through: Supernatant after overnight incubation with streptavidin magnetic beads; Eluate: Protein sample eluted with 40 μL of buffer; SA-HRP: Streptavidin labeled with horseradish peroxidase; Molecular mass markers (in kDa) are on the left side of each panel.

Figure S5

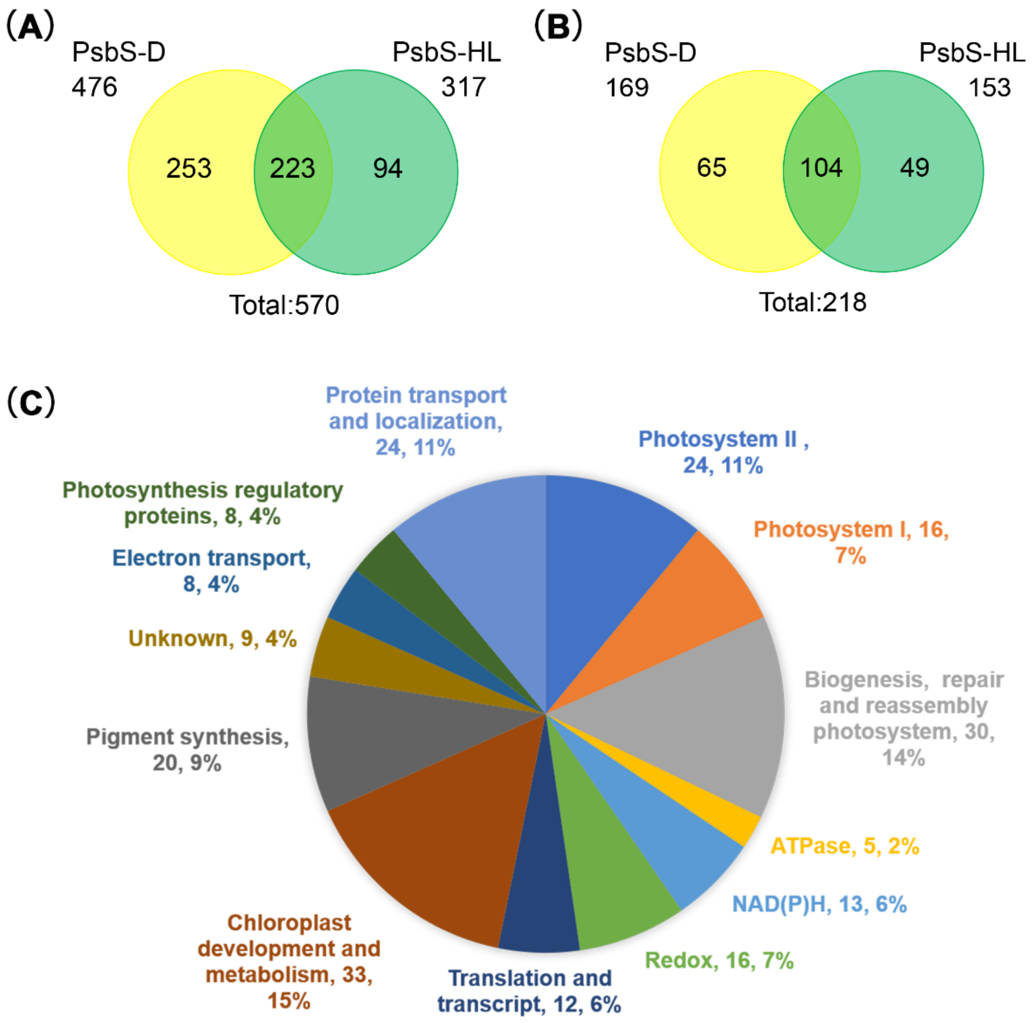

Figure S5 Proximity-labeled proteins associated with PsbS under dark and high light conditions.

(A and B) Venn diagrams separately depicting the biotinylated proteomes identified in PsbS-TurboID-eGFP (#18) transgenic plants under dark (PsbS-D) and high-light (PsbS-HL) conditions in chloroplast (A) and thylakoid (B). (C) Scatter plot depicting the relative differences (-$\log_{10}$ P-value) in chloroplast protein abundance between plants expressing PsbS-TurboID-eGFP under dark and high-light conditions.

Figure S6

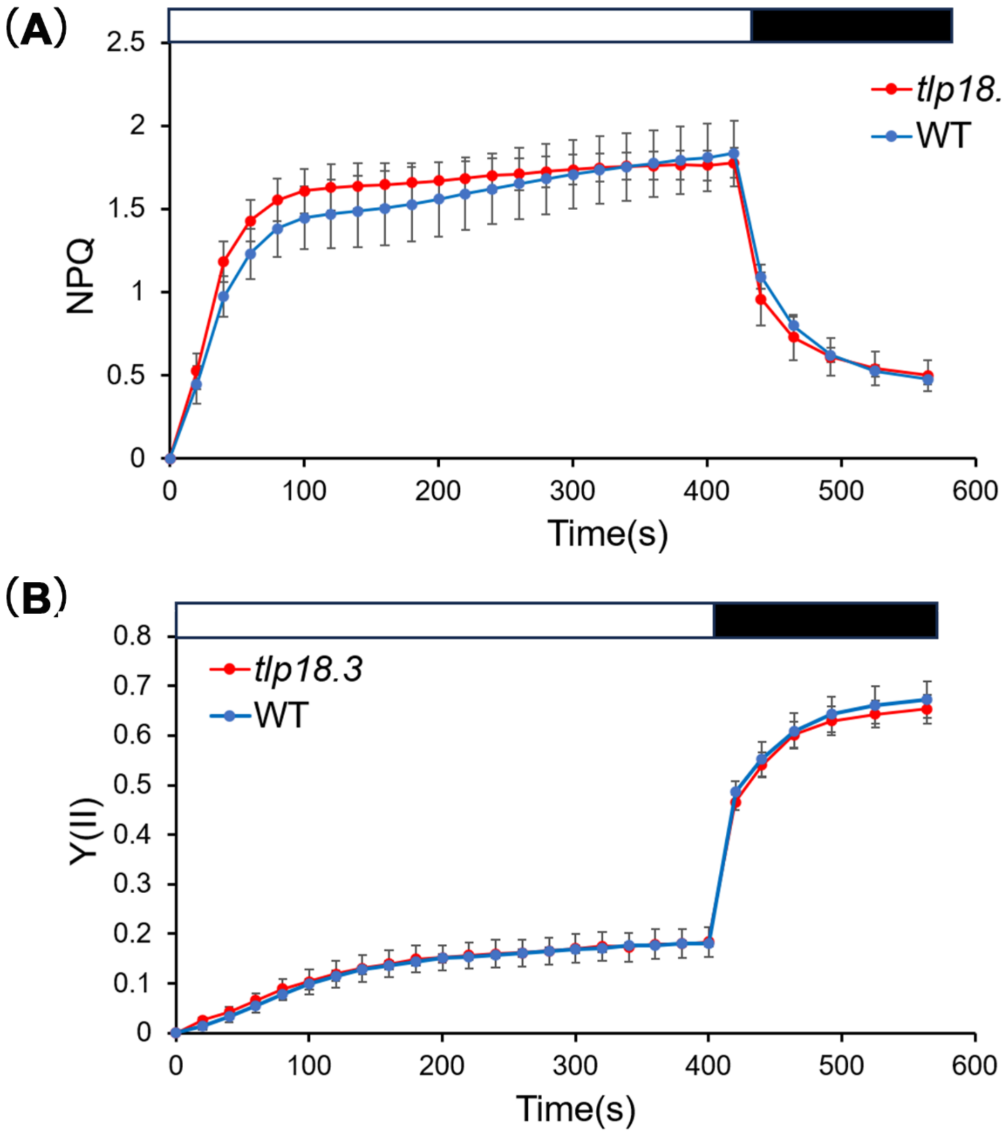

Figure S6 Determination of NPQ and Photosystem II (Y(II)) in the *tlp18.3* mutant. (A and B) Measurement of time-course-induced NPQ and Y(II) of the WT and *tlp18.3* mutant under 685 μmol·m^{-2}·s^{-1}. After a 2-hour dark treatment, light treatment was performed for 7 minutes (white box), followed by a 2-minute and 24-second dark treatment (black box) to relax qE. Data are presented as mean ± S.D. (n = 6).
